# A Systematic Review on Cross-Cultural Comparative Studies of Sleep in Young Populations: The Roles of Cultural Factors

**DOI:** 10.3390/ijerph18042005

**Published:** 2021-02-19

**Authors:** Mina Jeon, Dagmara Dimitriou, Elizabeth J. Halstead

**Affiliations:** Sleep Education and Research Laboratory, Department of Psychology and Human Development, UCL Institute of Education, London WC1H 0AA, UK; d.dimitriou@ucl.ac.uk (D.D.); l.halstead@ucl.ac.uk (E.J.H.)

**Keywords:** cross-cultural studies, culture, sleep duration, sleep disturbances, infant, toddler, children, adolescents

## Abstract

Recent studies have shown that sleep is influenced and shaped by cultural factors, including cultural values, beliefs and practices. However, a systematic understanding of how cultural factors in countries may influence sleep duration and sleep disturbances is still lacking. Therefore, we focused on a comparison of sleep duration and disturbances in young populations between countries. We report cross-cultural differences between the child, parent and environmental factors, and their association with sleep duration and disturbances. The review is based on literature searches of seven databases published until December 2020. Studies were included if they investigated sleep duration and disturbances of individuals up to 18 years across at least two or more countries. The results of this review have shown that sleep duration and disturbances vary between countries and regions and certain factors (e.g., bedtime routines, sleeping arrangement, physical activity and psychological functioning) have been associated with sleep duration or disturbances. This review also demonstrates that certain factors which were associated with sleep duration or disturbances in one country, were not shown in other countries, suggesting a need for recommendations for age-related sleep duration and sleep interventions to consider cultural differences that influence sleep duration or disturbances in individual countries or regions.

## 1. Introduction

Throughout the decades, there has been a growing body of literature that recognises the importance of sleep in several areas of child development, including physical, psychological and cognitive development [1,2,3,4]. Children and adolescents spend most of their time learning, therefore it is important to consider the impact of poor sleep on cognitive development, which could influence children’s academic performance [1]. Specifically, insufficient sleep and poor sleep quality can cause daytime sleepiness, which, in turn, can have a negative effect on attention and learning motivation, thus resulting in poorer academic performance [5]. A recently published paper found a benefit of midday napping on academic performance [6]. Liu and colleagues (2019) examined midday napping and sleep duration in approximately 3000 fourth, fifth and sixth graders aged between 10 and 12 years old in China. It was found that children who napped 3 times or more per week, or longer than 31 min on average, demonstrated up to a 7.6% increase in academic performance [6].

The literature on sleep in children and adolescents has highlighted the importance of achieving adequate sleep duration and quality. As children develop, the recommended amount of sleep changes. According to the National Sleep Foundation’s guideline for age-appropriate sleep duration, 14 and 17 h of sleep is recommended for newborns, followed by 12 and 15 h for infants, 11 and 14 h for pre-schoolers, 9 and 11 h for school-aged children and 8 to 10 h for adolescents [7]. However, most recent research has indicated that younger children are commonly reported to have a shorter sleep duration than the currently recommended average sleep duration for their age group [7,8,9]. In addition, the vast majority of studies have found that sleep disturbances are present in childhood and adolescence [10,11,12]. Taken together, it is crucial to establish factors that may impact on sleep duration and contribute to sleep disturbances in young populations. Recent studies have shown evidence that sleep is also influenced and shaped by cultural factors, including cultural values, beliefs and practices [13,14,15]. Previous studies have typically either focused on differences in sleep patterns between two or more countries [16,17] or have focused on sleep patterns between different races, such as White vs. Hispanic, within one country, region or geographical area [18]. A systematic understanding of how cultural values, beliefs and practices from different countries contribute to sleep duration and disturbances is still lacking. Most cross-cultural studies to date have focused on self-report or proxy report of behavioural sleep problems. Therefore, in the current systematic review the term “sleep disturbances” is used to describe a range of behavioural sleep issues, i.e., bedtime resistance, sleep latency, night awakening and daytime sleepiness.

This review aimed first to report the differences identified in sleep duration and disturbances in infants, pre-school aged children, school-aged children and adolescents between two or more countries, and second to report the differences in child, parent and environmental factors and their association with children’s sleep duration and disturbances across countries. Finally, we will identify and discuss the role of cultural factors associated with sleep. These are the child, parent or environmental factors that have been identified in previous literature as being influenced by the culture, values or beliefs of an individual or society within a country and discuss their influence on children’s sleep duration and disturbances.

## 2. Materials and Methods

### 2.1. Search Strategy

This systematic review followed the reporting guidelines and criteria of Preferred Reporting Items for Systematic Reviews [19]. Articles were searched using seven electronic databases: Pubmed, Embase, Web of Science, Ovid Medline, PsycInfo, Cochrane Library and Scopus. This search agglomerated all publications up to December 2020 using the following search terms: (“sleep”[title]) AND (“infant* OR toddler* OR child* OR adolescen* OR youth OR young”[title/abstract]) AND (““cross cultur*” OR cross-cultur* OR cultur*”[title/abstract]). This search produced 1213 articles. Citations were downloaded into Endnote software [20] and duplicates were removed. The search was restricted to articles published in English and peer-reviewed journals. No publication date restriction was applied. This review focused on peer review journal articles, and therefore grey literature was not searched; however, the reference lists of included papers and Google Scholar were manually searched to identify further relevant publications (*n* = 3).

### 2.2. Selection Criteria

Each study was screened to meet all the inclusion criteria and none of the exclusion criteria. The inclusion criteria were (1) either an observational or experimental study using a quantitative or qualitative approach, (2) included a comparison of populations between two or more countries (3) included one or more measures of sleep duration (i.e., bedtime, wake up time, nocturnal sleep duration, habitual naps and total sleep time) and/or sleep disturbances (i.e., nocturnal awakenings, bedtime resistance, sleep latency, sleep problems, daytime sleepiness and sleep quality) and (4) participants aged <18 years old. Exclusion criteria were (1) no measures of sleep duration or sleep disturbances (2) other types of articles (e.g., review papers, commentary, a case study and conference abstracts), and (3) use of secondary data with no additional analysis, or with similar aims to a previously published article with primary data. If multiple studies analysed the same dataset, studies were only included if they investigated different outcomes.

### 2.3. Study Screening and Data Extraction

The title and abstract of all identified papers were screened for relevance. The full texts of potentially relevant articles were read in full to assess inclusion eligibility. Reviewers (MJ and EJH) independently assessed relevant articles based on the predefined inclusion/exclusion criteria, this was then cross-checked. Cohen’s κ was conducted to determine the inter-rater agreement at the title and abstract screening stage and the full text screening stage. There was good agreement between reviewers at the title and abstract screening κ = 0.638, *p* < 0.001 and very good agreement at the full text screening stage k = 0.935, *p* < 0.001. Any discrepancies shown in the full text screening stage was resolved by the third reviewer (DD) and a final list was agreed. Meta-analyses were not conducted as the results were not found to be sufficiently homogenous in terms of statistical, clinical and methodological characteristics.

### 2.4. Quality Assessment

The current review focused on studies that compared children’s sleep patterns across two or more countries, and thus the methodological quality of all included studies was analysed using a case–control Critical Appraisal Skills Programme (CASP) tool [21], which has been used in previous systematic reviews [22]. Two reviewers assessed the methodological quality of included studies based on the following 12 criteria: (1) clearly focused question, (2) appropriate design, (3) clearly defined recruitment, (4) sample size based on a power calculation, (5) outcome measurements valid and reliable (defined as validated measures to be used in children and adolescents), (6) exposure measurements valid and reliable, (7) confounding factors accounted for, (8) appropriate statistical analysis, (9) precise estimate of effect, (10) reliable results with acknowledged possible bias, (11) ability to generalise results and (12) interpretation related to the existing evidence. Each paper was assigned to be low (≤8), moderate (≤10) or high quality (≤12) depending on the number of criteria they have met and any discrepancies were resolved by consensus. The results of the quality assessment process are listed in Appendix A.

Overall, cross-cultural studies gathered in this review displayed moderate methodological quality. However, of the 25 identified studies, eight studies were assigned to be low quality [16,23,24,25,26,27,28,29]. Six of these received low quality ratings due to low statistical power in participant numbers and the use of questionnaires which had not been previously validated to examine child, parental and environmental factors [23,24,25,26,28,29]. However, it should be noted that of these eight studies, six were included in the review to represent comprehensive understanding about the previous cross-cultural studies on sleep duration and disturbances in young population. The other two studies with low quality [16,27] were excluded from this review as these studies met less than 6 criteria of the quality assessment. Therefore, these studies would have compromised the quality of the current review as appropriate statistical analysis to examine differences in sleep duration and disturbances across countries was not conducted, and the studies did not use validated sleep measures.

### 2.5. Search Results

**Figure 1:**
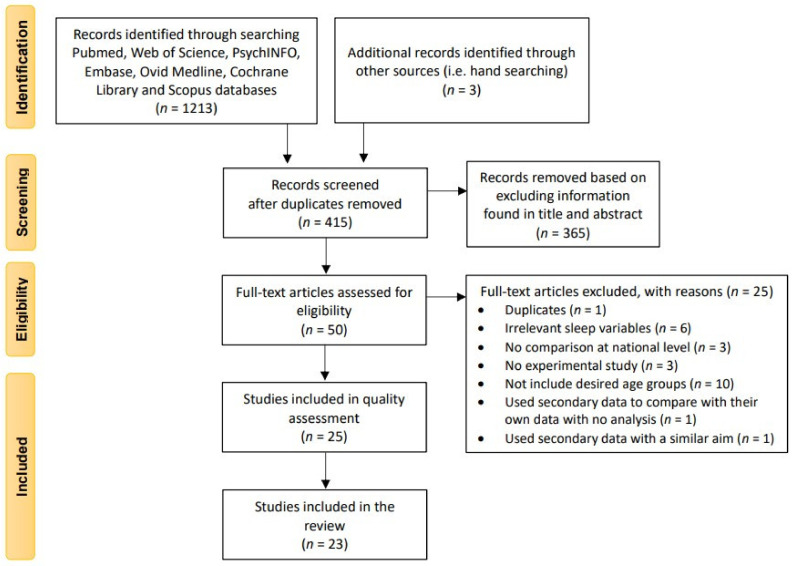
Flow diagram for the systematic search procedures.

The screening process is presented as a flow diagram (Figure 1). Our electronic and manual search strategy resulted in 415 unique citations after removal of duplications (*n* = 801). From these citations, we identified 50 for further evaluation based on information provided in the title and abstract. A total of 25 (50%) studies met inclusion criteria after full-text review.

## 3. Results and Discussion

### 3.1. Sleep Duration and Disturbances

#### 3.1.1. Summary

Thirteen studies compared sleep duration across 40 countries, see Table 1 for characteristics of included studies relating to sleep duration variables, including age, gender, sleep measurement, sleep duration and a brief summary of findings [17,25,30,31,32,33,34,35,36,37,38,39,40]. Fourteen studies compared sleep disturbances across 34 countries, see Table 2 for characteristics of included studies relating to sleep disturbance variables, including age, gender, sleep measurement, sleep disturbance and a brief summary of findings [17,23,25,29,30,31,33,34,36,37,38,39,40,41]. All studies used parent-report or self-report questionnaires to measure sleep, with the exception of two studies [25,37] that used actigraphy watches in addition to parent- or self-report questionnaires. See Box 1 for a summary of key findings for sleep duration and sleep disturbances.

Box 1 **Key Findings for sleep duration and sleep disturbances.**

- In general, children from Europe, North America and Australasia were more likely to have an earlier bedtime, earlier wake up time and longer nocturnal sleep duration than children in Asia and the Middle East region.
- Infants in Asia were more likely to have more frequent and longer habitual naps than other countries.
- Parents from countries in Asia reported more sleep disturbances in their infants and children than those from Europe, North America and Australasia, including nocturnal awakenings, bedtime resistance, sleep latency and general sleep problems.
- Cultural differences in sleep duration and disturbances were found between countries within the same region, however results were varied.
- Most studies used parent-reported questionnaires (Children’s Sleep Habits Questionnaire; CSHQ and the Brief Infant Sleep Questionnaire; BISQ).
- Discrepancies were identified between actigraphy and parent/self-report sleep data for nocturnal sleep duration and sleep quality.
- Most studies included typically developing children, one study included children with Autism Spectrum Disorder (ASD).

#### 3.1.2. Sleep Duration: Bedtime, Wake Up Time, Nocturnal Sleep Duration, Habitual Naps and Total Sleep Time

Several studies found significant differences in children’s bedtime [17,31,34,36,37,38,39,40], wake up time [17,31,34,35,38,39,40], nocturnal sleep duration [17,31,32,35,36,38,40], total duration and frequency of habitual naps [17,30,33,34,36,38,40] and total sleep time [17,34,36,38,39]. 

• Countries in the Middle East region vs. other countries.

Countries in the Middle East region reported the latest bedtime and wake up time, and the shortest nocturnal sleep duration and 24-h total sleep time when compared to all other countries [38].

• Countries in Asia vs. Europe, North America and Australia.

Countries in Asia reported a later bedtime [17,34,36], later wake up time [17,34], shorter nocturnal sleep duration [17,36], more frequent and longer duration of habitual naps [17,34] and shorter 24-h total sleep time [17,34,36] than countries in Europe, North America and Australasia. 

• Countries within Asia.

Results were varied in studies comparing countries within Asia, for example, one study compared infants in Japan with infants from 11 other countries in Asia and found no significant differences in bedtime, wake up time, nocturnal sleep duration and total sleep time [33]. However, another study found pre-school aged children in China had a significantly later bedtime and wake up time and longer total sleep time than preschool aged children in Japan [39]. Within Asia, significant differences were found in both the frequency of naps and the total duration of time spent napping during the day in infants and pre-school aged children [33,36,40]. For example, infants in Japan had less frequent and a shorter duration of habitual naps than infants in 11 other Asian countries [33] and infants in South Korea had less frequent and a shorter duration of habitual naps compared to infants in other Asian countries [36].

• Countries within Europe.

Nocturnal sleep duration in children aged between 2 and 9 years in eight European countries ranged from 9.5 h to 11.2 h. Children in Northern Europe (including Sweden, Germany and Belgium) had a significantly longer nocturnal sleep duration than children in Southern Europe (including Italy, Spain and Cyprus) and Eastern Europe (including Estonia and Hungary) [32].

• U.S.A. vs. Australia, Italy and China.

Adolescents in the U.S. were more likely to have earlier wake up time and shorter nocturnal sleep duration than adolescents in Australia [35]. However, no significant difference was found in bedtime between adolescents in the U.S. and Australia. Adolescents in the U.S. were reported to have more naps (>1 h in duration) in the last month when compared with adolescents in Italy [30]. By contrast, children in the U.S. had a significantly earlier bedtime, later wake up time and longer nocturnal sleep duration than children in China [31].

• Australia vs. Canada, Netherlands

Children and adolescents in Australia were found to have a significantly longer nocturnal sleep duration as measured by subjective sleep measures (e.g., sleep diary and questionnaire) when compared with adolescents in the Netherlands [25] and children in Canada [37]; however, when comparing the actigraphy data, differences in nocturnal sleep duration were not found [25,37]. Furthermore, actigraphy report found children in Australia has a significantly earlier bedtime and later wake up time than their peers in Canada [37].

#### 3.1.3. Sleep Disturbances: Nocturnal Awakenings, Bedtime Resistance, Sleep Latency, Sleep Problems, Daytime Sleepiness, and Sleep Quality

Several studies found significant differences in child nocturnal awakening frequency [17,23,30,31,33,34,36,38,39,40], nocturnal awakening duration [17,33,34,36,38,40], bedtime resistance [17,23,30,31,33,38,39], sleep latency [17,29,30,34,39,40], overall sleep problems [17,23,31,33,34,38,40], daytime sleepiness [31] and sleep quality [17,25,30].

• Countries in the Middle East region vs. other countries.

Countries in the Middle East region reported the highest frequency and the longest duration of nocturnal awakenings, and the highest percentage of bedtime resistance when compared to all other countries [38]. Countries in the Middle East region also reported the higher rate of sleep problems when compared to countries in Europe, North America and Australasia; however, countries in the Middle East region reported a lower rate of sleep problems overall when compared to countries in Asia [38].

• Countries in Asia vs. Europe, North America and Australia.

Countries in Asia reported greater problems with the frequency and duration of nocturnal awakenings [17,36], sleep latency [17,34], sleep quality [17] and overall sleep problems [17,34] than countries in Europe, North America and Australasia. Conversely, study [34] found no significant difference in either the frequency or total duration of nocturnal awakenings. In addition, no significant difference was found in daytime sleepiness [34] or bedtime resistance [17] between countries in Asia vs. Europe, North America and Australia. One study found that infants in South Korea had a shorter duration of nocturnal awakenings than infants in Europe, North America and Australasia [36].

• Countries within Asia 

Infants and pre-school-aged children in Japan had significantly less problems with nocturnal awakenings, although infants in Japan were reported to have more problems with bedtime resistances when compared to infants in 11 other Asian countries [33] and children in China [39]. One study found greater problems with nocturnal awakenings and overall sleep disturbances in children with ASD in Indonesia than children with ASD in Japan [41]. However, no significant differences were found for bedtime-related sleep problems [41]. Significant differences in the frequency and duration of nocturnal awakenings were found in studies comparing six Southeast countries [40] and comparing South Korea vs. other countries in Asia [36]. For example, infants in South Korea had less frequent nocturnal awakenings and a shorter duration of nocturnal awakenings than infants in other countries in Asia. 

• U.S.A vs. Italy, Japan and China.

Several studies compared the U.S. and other countries (e.g., Japan, Italy and China) and found significant differences in nocturnal awakenings [23,30,31], bedtime resistance [23,30,31], sleep latency [30], overall sleep problems [23,31], daytime sleepiness [31] and sleep quality. Infants in the U.S. had significantly greater problems with nocturnal night awakenings, bedtime resistance and sleep problems compared to infants in Japan [23]; however, children in the U.S. had significantly less problems with nocturnal awakenings, bedtime resistance, daytime sleepiness and sleep problems compared to infants in China [31]. Furthermore, adolescents in the U.S. reported more problems with nocturnal awakenings, bedtime resistance and sleep latency than adolescents in Italy [30].

• Australia vs. Canada and Netherlands.

Studies that compared school-aged children and adolescents in Australia vs. school-aged children in Canada [37], and adolescents in the Netherlands [25] found no significant differences in the duration of nocturnal awakenings and sleep latency. Two studies compared children or adolescents’ sleep quality between countries [25,37]; however, a significant difference was only found in one study, this was between sleep quality in adolescents in Australia vs. adolescents in the Netherlands [25]. However, it should be noted that adolescents in Australia were found to have a significantly higher sleep quality than adolescents in the Netherlands when measured by sleep diary; however, when comparing the actigraphy data, higher sleep quality was found in adolescents in the Netherlands [25].

• Netherlands. Armenia vs. Indonesia

A greater number of school-aged children in the Netherlands had sleep latency problems (e.g., difficulty falling asleep) when compared with their peers in Armenia and Indonesia [29].

### 3.2. Cultural Factors: Child, Parent and Environmental Factors Associated with Sleep

#### 3.2.1. Summary 

**Figure 2:**
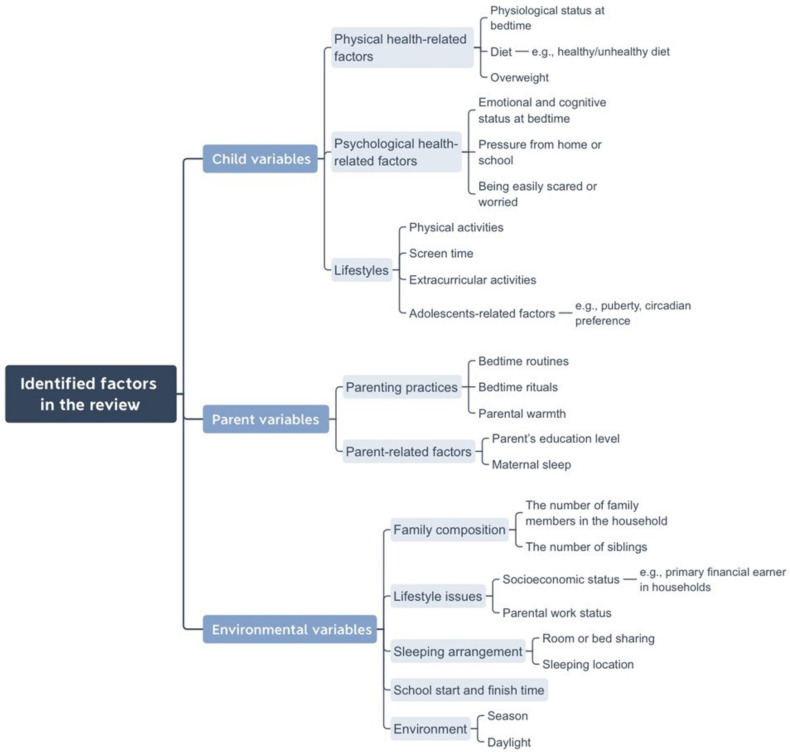
Parent, child and environmental factors identified in the review literature.

The included studies examined cross-cultural differences in child, parent and environmental factors across 51 countries (*n* = 19) [17,23,24,26,28,29,30,32,33,34,35,37,38,39,40,42,43,44,45] and investigated the association between these factors and sleep duration or disturbances (*n* = 15) [17,23,24,26,28,29,30,32,34,35,38,42,43,44,45]. See Table 3 for cultural factors examined with sleep duration or sleep disturbances, including age, gender and variables under the child, parental and environmental domains (see Appendix B for a full information on identified studies). We categorised cultural factors into the following headings and subheadings based on Owens’ study (see Figure 2) [46]. Parent’s education level was considered a parental variable as it could be an indication of parental knowledge on children’s development. Parental employment status was considered as an environmental factor as it may affect the amount of parental presence in the home environment. See Box 2, Box 3 and Box 4 for summaries of key findings for child, parental and environmental variables associated with sleep.

#### 3.2.2. Methodological Variability of Studies and the Identification of Culturally Relevant Factors

Studies used four different analysis approaches when assessing the relationship of child, parent and environmental factors with sleep duration and disturbances: (1) Two studies controlled for environmental factors (e.g., sleeping arrangement) when examining the differences in sleep duration or disturbances across countries [17,34]. (2) Two studies examined the relationship between cultural factors (e.g., parent-set bedtime and screen time) and sleep duration or disturbances after controlling for other cultural factors, including living in either country [32,35]. (3) Four studies examined the overall association between parental factors (e.g., bedtime routine and maternal sleep) and sleep duration or disturbances [34,38,44,45]. (4) Eight studies compared the relationship between specific factors and sleep duration or disturbances in each country [23,24,26,28,29,30,42,43]. 

Findings from eight studies demonstrated that some child, parental and environmental factors could be considered as culturally relevant factors. For example, a study examined the relationship between bed or bedroom sharing and the success in maintaining and reinitiating sleep in adolescents in the U.S. and Italy; this study found a significant relationship in adolescents in the U.S, however, not in adolescents in Italy [30]. These findings therefore can be interpreted as specific to the U.S. 

Child factors included psychological health-related factors (e.g., pressure from home and being easily scared), physical activity, and screen time. Parental and environmental factors included parental education level, presence of siblings, maternal education level and sleeping arrangement, all of which were found to be culturally relevant factors associated with children’s sleep duration or disturbances. The remaining factors were considered as universal factors, i.e., factors that were found to be associated with sleep duration or disturbances in multiple countries or regions (see Table 4 for a summary of the findings). However, note that most results were derived from a single study, therefore interpretation of the findings should be cautious and more cross-cultural studies are encouraged. 

#### 3.2.3. Child Variables Associated with Sleep

Box 2 **Key Findings for child variables associated with sleep.**

- Universal factors included unhealthy diet, poor emotional and cognitive status, and a preference for eveningness, which were all negatively associated with child and adolescent sleep duration and disturbances consistently across multiple countries.
- Cultural factors included poor psychological health (e.g., pressure from home and being easily scared), physical activities and screen time, and these were all negatively associated with child and adolescent sleep duration and disturbances and differed between countries.
- Studies that controlled for other factors (e.g., country, time spent playing outdoors and extracurricular activities) when examining the relationship between child factors and sleep duration or disturbances, did not find significant relationships between nocturnal sleep duration, child weight, and extracurricular activities.
- Physical health-related factors: physiological status at bedtime, diet and obesity.

Adolescents in Italy had better physiological status at bedtime (i.e., fewer children were engaging in physiologically activating behaviours) than adolescents in the U.S. [30]. However, in adolescents in both Italy and the U.S. the study found no significant relationship between physiological status at bedtime and sleep quality [30].

In 12 countries in Europe, South and North America, Australasia, Africa and Asia, school-aged children who had an unhealthy diet were more likely to have a later bedtime, a shorter sleep duration, and worse sleep quality, whereas children who had a healthy diet were more likely to have an earlier bedtime [43]. This relationship was found across all 12 countries [43]. Furthermore, in general, being overweight was significantly associated with a longer nocturnal sleep duration in children from eight European countries. However, further analysis reported this finding was not significant after controlling for the effect of other variables (e.g., age, parental education level, country, season and daily light hours) [32].

Based on similar findings across all 12 countries, diet could be determined as a universal factor associated with sleep duration and disturbances. This finding is supported by previous research which demonstrated children who snack in-between meals or after dinner were found to have decreased sleep duration and quality [47]. In addition, the discomfort from indigestion due to an unhealthy diet is likely to disturb sleep [48].

• Psychological health-related factors: emotional and cognitive status at bedtime, pressure from home or school and being easily scared or worried.

In a study comparing adolescents in the U.S. and Italy, a significantly better emotional and cognitive status at bedtime was observed in Italy. However, in both countries adolescents who had a more positive emotional and cognitive status at bedtime had better sleep quality [30].

Home or school pressures, being anxious or worried, and the association with sleep disturbances was examined across school-aged children from the Netherlands, Armenia and Indonesia [29]. This study found that children in Armenia were the most likely to have home and school pressures and were feeling anxious, whereas children in Indonesia were most likely to be worried [29]. The relationships between home pressures, being anxious and sleep bruxism varied in each country [29]. For example, home pressure was only associated with sleep bruxism in children in the Netherlands and Indonesia, and not in Armenia. Feeling anxious was associated with greater sleep bruxism in children in the Netherlands and Armenia, however, no association was found in children in Indonesia [29].

• Lifestyles: physical activities.

In 17 countries in Europe, North America, Australasia and Asia, greater physical activity (e.g., daily outdoor time) of pre-school aged children were generally associated with a later bedtime, longer daytime sleep, and shorter nocturnal sleep duration [34]. Similarly, another study examined school-aged children from 12 countries in Europe, South and North America, Australasia, Africa and Asia. This study found that moderate-to-vigorous physical activity was associated with an earlier bedtime, a shorter nocturnal sleep duration, and poorer sleep quality [43]. However, different associations were found between each country, however, directions and details of these associations were not reported [43]. In contrast to this finding, one study examined pre-school-aged and school-aged children in eight European countries and found no significant association between physical activity (e.g., time spent playing outdoors) and nocturnal sleep duration; this finding was after controlling for factors such as age, parental education level, country, season, daylight hours, obesity, and screen time [32]. These inconsistent findings are in line with existing research results. In previous systematic reviews, a positive relationship between exercise/physical activity and sleep was reported in some studies [49,50], although in some studies negative relationship between physical activity and sleep duration was reported (e.g., shorter total sleep time was found in physically active children compared with less active children) [51,52]. These inconsistencies in results may be attributed to the different effect of physical activity on sleep in each country, thus a cultural factor.

However, these variations may also be caused by methodological issues of measuring physical activity. Previous studies have shown that the vigour of the exercise influences sleep in different ways [53]. Moderate physical activity appears to be more effective than vigorous activity in improving sleep. However, in the study included in this review, vigorous physical activity during the day was not explored in relation to sleep the following night [43].

• Lifestyles: screen time (watching tv, playing video games, and using computers).

In a study comparing Asia, Europe, North America and Australia, pre-school-aged children in Asia were more likely to have a longer total screen time than in Europe, North America and Australasia. In general, longer duration of screen time was associated with a later bedtime, later wake up time, longer sleep onset latency, more frequent night awakenings and nocturnal sleep duration [34]. However, a separate analysis for each region was not conducted. A study comparing Mozambique vs. Cape Verde examined the relationship between screen time and sleep disturbances in each country and found different associations in each country [28]. Frequency of watching TV at bedtime was associated with greater sleep disturbances in pre-school-aged and school-aged children from Mozambique, although not in children from Cape Verde [28]. Furthermore, two studies controlled for other factors when examining the relationship between screen time and sleep duration [32,43]. Though a study investigating pre-school-aged and school-aged children in eight European countries found no significant association between screen time and sleep duration after controlling for other factors (e.g., age, parental education level, country, season, daylight hours and overweight) [32], a study investigating school-aged children from 12 countries in Europe, South and North America, and Asia found significant associations between screen time and bedtime after controlling for factors such as age, highest parental education and body mass index [43]. Therefore, the reported association between screen time and bedtime could be a true cultural association, and these associations were found to be different between countries (however, specific data were not reported) [43].

Overall, the results of screen time on sleep duration or disturbances in children varied between countries [28,43]. This could be explained by the previous findings reporting that the effect of screen time on sleep could vary depending on the availability of devices in the bedrooms [54], in addition to parental control which can have a moderating role between bedtimes and watching TV [55]. Therefore, it is important to consider other factors may be influencing this association that is specific to these countries.

• Lifestyles: academic-related factors (extracurricular activities).

Extracurricular activities were only investigated in one study comparing adolescents in the U.S. and Australia [35]. The study found that adolescents in the U.S. were more likely to spend time on extracurricular activities than adolescents in Australia [35]. A significant relationship was found between the duration of extracurricular activities and sleep duration, specifically, adolescents had 4 minutes less sleep for every hour spent on extracurricular activities; however, after controlling for the effect of living in either country to account for cultural differences, the significant effect of extracurricular activities on sleep duration was not maintained [35].

• Lifestyles: adolescent-related factors (puberty and circadian preference).

Puberty and circadian rhythm were only examined in two studies [30,35]. Adolescents’ circadian preference was compared in two studies (e.g., the U.S. vs. Italy [30] and the U.S. vs. Australia [35]) and no significant differences were found. The relationship between circadian preference and sleep quality was examined in one study and this study found that morningness (a preference for waking up early and going to bed early) was associated with significantly better sleep quality than eveningness (a preference for staying up late and waking up late) in both adolescents in Italy and the U.S. [30]. This may be due to adolescents naturally delaying their sleep times due to developmental changes in their circadian rhythms regardless of the country they live in [56,57].

Students’ earlier school start time causes insufficient sleep and excessive daytime sleepiness in adolescents [58,59]. However, earlier school start times may have had less of an impact on adolescents who were morning-oriented, relating to a preference to sleep early and wake up early. Furthermore, it is unclear if sleep quality is also affected by social factors (such as alcohol intake) related to adolescent’s preference for staying up late (i.e., evening-oriented). Note that there were only two countries exploring this factor with limited countries for comparison.

#### 3.2.4. Parent Variables Associated with Sleep

Box 3 **Key Findings for parental variables associated with sleep.**

- Universal factors included bedtime routine, parental warmth, and maternal sleep which were all positively associated with children and adolescents’ sleep duration and disturbances consistently across multiple countries.
- Cultural factors included parental education level which was associated with parent’s perception on children’s sleep disturbances and differed between countries.
- Parenting practices: bedtime routines, bedtime rituals and parental warmth.

In studies comparing Europe, North America and Australia vs. Asia or Middle East region, there was a consensus that infants and pre-school-aged children from Europe, North America and Australasia have a more consistent bedtime routine than infants from Asia and the Middle East region [34,38]. However, one study initially showed a more consistent bedtime routine in infants from Asia than in Europe, North America and Australasia [17], conversely in their Corrigendum, the results became consistent with other studies [60]. Studies also found that bedtime routine was associated with earlier bedtime and wake up time, shorter sleep latency, fewer and shorter night awakenings, shorter duration of habitual naps and longer nocturnal sleep [34,38,44]. However, these studies did not report the associations specifically in each country. Consistent bedtime routines were also different between countries in Asia [33,40]. For example, infants in Japan were more likely to have more consistent bedtime routine than infants from other countries in Asia [33]; although, these studies did not examine the association between bedtime routine and sleep duration or disturbances [33,40]. Two studies comparing adolescents in the U.S. vs. adolescents in Italy and Australia found no associations between bedtime routine and sleep quality in both adolescents in the U.S. and Italy [30]; however, the significant impact of parent-set bedtime on sleep duration of adolescents was found after controlling for the effect of living in either country (U.S. vs. Australia) [35]. Therefore, the parental factor of bedtime routine leading to parent-set bedtime appears to be a universal factor. Note that these studies did not take into account common activities in bedtime routines which could be varied across countries, seemingly influenced by cultural values. For instance, a study investigated racial/ethnic differences in bedtime routine activities and found that African American families were less likely to engage in interactive (e.g., parent-child reading, singing or other interactions) and hygiene-related routines (bathing and/or brushing teeth) than Caucasian families [61]. Furthermore, parents in Europe, North America and Australasia were more likely to adopt independent sleep initiation methods (e.g., sleeping in a crib alone) at an earlier age [62]. However, as this study did not investigate the association between sleep initiation or resuming methods and children’s sleep duration or disturbances, it is difficult to conclude which methods need to be encouraged for better sleep of infants and children.

Bedtime rituals such as parent sleep initiating practices (e.g., bottle-feeding, nursing and rocking) and sleep resuming strategies (e.g., picking up and rub or pat in crib/bed) were examined in two studies comparing the U.S. vs. Japan [23], and Europe, North America and Australasia vs. Asia [42]. Parents of infants in the U.S. were more likely to have body contact when infants woke up at night than parents of infants in Japan, however no significant differences were found in bottle-feeding to initiate sleep of their infants [23]. In contrast, another study found that parents in Asia were more likely to use bottle-feeding, nursing and rocking methods to initiate their infant’s sleep than parents in Europe, North America and Australasia [42]. Similarly, parents in Asia were more likely to nurse or play/sing with their infants to resume their sleep, whereas parents from Europe, North America and Australasia were more likely to have body contact or bring a child to parent’s bed and encourage infants to resume their sleep by themselves (e.g., allowing them to cry andfall asleep) [42]. However, no significant differences were found for watching TV or video as sleep initiating and resuming methods [42]. These two studies only compared the differences in bedtime rituals between countries or regions and did not examine the association between bedtime rituals and sleep duration or disturbances.

Parental warmth (e.g., positive parenting) was examined in one study between adolescents in Switzerland and Georgia found that parental warmth was significantly associated with better sleep quality and longer sleep duration in both countries [26].

• Parent-Related Factors: Parent’s Education Level and Maternal Sleep

The majority of studies only compared differences in parental education level across countries [17,23,24,28,32,34,37,39,45]. Studies comparing maternal education level between Europe, North America and Australasia, and Asia showed a higher number of mothers in Asia completed higher education (e.g., undergraduate and postgraduate degrees) than in other countries [17,45]. Within Asia, both mothers and fathers in China were more likely to have a higher education level than mothers and fathers in Japan [39]. In Africa, both mothers and fathers in Mozambique were more likely to have more than 10 years of education than parents in Cape Verde [28]. Three studies found non-significant differences in maternal education level comparing parents in Europe, North America and Australasia vs. Asia, Australia vs. Canada, and Japan vs. the U.S. [23,34,37]. However, fathers in Japan had significantly higher level of education compared with fathers in the U.S. [23].

The associations between parental education level and children’s sleep duration or disturbances were examined in three studies, although these results are limited [24,28,32]. A study comparing countries in Europe, North America and Australasia vs. Asia found that higher maternal education was associated with more parent-reported severe sleep disturbances (not specified) in children in Asia; however, the same association was not shown in Europe, North America and Australasia [24]. A study investigating countries in Africa found that higher maternal and paternal education was associated with less parent-reported sleep disturbances (not specified) in children; however, the association between maternal education and parent-reported sleep problems was only shown in mothers in Cape Verde, whereas the association between paternal education and parent-reported sleep problems was only shown in fathers in Mozambique [28]. Furthermore, a study controlled other factors (e.g., age, country, overweight, screen time and time playing outside) when investigating the relationship between medium or low parental education and nocturnal sleep duration of children in eight European countries found no significant relationship [32].

One study examined maternal sleep in relation to their children’s sleep across 13 countries in Europe, North America, Australasia and Asia, and found that, overall, maternal sleep duration and disturbances were found to be strongly linked with their child’s sleep duration and disturbances, and mothers were more likely to report that their child’s sleep pattern affects their sleep when their child is young compared with when their child is older [45]. Better maternal sleep seems to be associated with children’s sleep duration or disturbances in multiple countries, however further studies should conduct a separate analysis for each country to confirm this finding.

#### 3.2.5. Environmental Variables Associated with Sleep

Box 4 **Key findings for environmental variables associated with sleep.**

- The number of family members was associated with children and adolescents’ sleep duration and disturbances across multiple countries.
- Cultural factors included presence of siblings, co-sleeping or falling asleep alone, and maternal employment status, which were all associated with children and adolescents’ sleep duration and disturbances and differed between countries.
- Studies that controlled for other factors (e.g., country, time spent playing outdoors and extracurricular activities) when examining the relationship between environmental factors and sleep duration, did not find significant relationships between nocturnal sleep duration, and school start/finish time, season and daylight duration. This may represent that the effect of other factors (e.g., country) are significant in these relationships.
- Family composition: the number of siblings and family members in one household.

Cultural differences in the number of siblings in a household were examined in three studies [28,39,45], and significant differences were shown in two studies [28,39]. Pre-school-aged children in Japan were more likely to have more siblings compared with their peers in China, and school-aged children in Cape Verde were more likely to have three or more siblings than school-aged children in Mozambique [28,39]. The impact of the presence of siblings (only child vs. not only child) on parent-reported sleep disturbances in infants was examined in one study and found that children who have siblings in Asia were more likely to be defined as having severe sleep disturbances (unspecified) compared with children who had no siblings; however, this was not the case for children in Europe, North America and Australasia [24]. This is possibly due to infants or toddlers in Asia more commonly sharing beds or rooms with their siblings; therefore, their sleep may be disturbed by their siblings. Another reason may be related to mothers of infants or toddlers in Asia providing various sleep initiation methods [42] to manage the sleep of two or more children, this in turn may cause poor maternal sleep, and bias mothers to perceive that their child has more severe sleep problems. However, these findings suggest the presence of siblings is a cultural factor.

Cultural differences in the number of family members in a household were examined in three studies [23,28,39] and two studies did not find any significant differences (e.g., Japan vs. China and Japan vs. U.S) [23,39]. One study found a significantly higher number of family members (over 5) in one household of school-aged children in Cape Verde compared with Mozambique [28]; however, the association between having more family members in one household and parent-reported sleep disturbances was not significant [28].

• Lifestyles: socio-economic status and parental work status.

One study compared socioeconomic status between countries and found the primary financial earner in households of adolescents in America was significantly more likely to have a professional occupation compared with the primary financial earner in adolescents in Italy [30]. No significant association between socioeconomic status and sleep quality was found in adolescents in Italy vs. the U.S. [30].

Cultural differences in maternal employment status were examined in four studies [17,23,34,45]. Although a significant difference in the percentage of employed mothers was not found between in Japan and in the U.S. [23], parents in Asia were more likely to have full-time jobs than those in Europe, North America and Australasia [17,34,45]. One study found that mothers in Asia with a higher employment level were significantly more likely to define children’s sleep as severe sleep disturbances, conversely this was not found in mothers from Europe, North America and Australasia [24]. These results have suggested that maternal employment level is a culturally relevant factor associated with parent-reported severe sleep disturbances of children in Asia, although not for children in Europe, North America and Australasia. No studies investigated the association between maternal employment level or socio-economic status and sleep duration.

• Sleeping arrangement: room or bed sharing and sleep location.

Sleeping arrangement was the most frequently examined environmental factor (*n* = 7) [17,23,30,33,34,40,42]. Infants in Europe, North America and Australasia were less likely to share a room or bed with parents than those from Asia [17,34]. These differences in co-sleeping between countries could reflect culturally based values and beliefs. Previous research has shown that co-sleeping is promoted in cultures where interdependency and parent–child bond are the goals in the child-rearing practices [63,64,65]. Conversely, co-sleeping is discouraged in cultures where individualism and autonomy are valued and when it is believed that co-sleeping in hazardous environment might increase the risk of infant mortality rates [64,66]. Thus, lower rates of co-sleeping found in Europe, North America and Australasia could be due to their cultural value of independent sleeping and the view that co-sleeping can be a harmful environment [65]. Whereas higher rates of co-sleeping found in Asia could be due to their cultural value of interdependency and co-sleeping increases the parent-child bond. Furthermore, co-sleeping or falling asleep alone was found to be a cultural factor associated with sleep duration and disturbances in infants from Europe, North America and Australasia, however not for infants from Asia [42]. For example, infants in Europe, North America and Australia who were frequently falling asleep alone in own crib/bed had a longer nocturnal sleep duration, less night awakenings, and longer episodes of continuous sleep [42]. However, the same association was not shown in infants from Asia [42]. This could also explain as reasons as why parents in Europe, North America and Australasia tend to avoid co-sleeping [42].

Cultural differences in sleeping arrangement were also found within the same region. For example, variability was shown in the percentages of children sleeping in own room [33,40] or sharing bed/bedroom with parents [40] within countries in Asia. This finding is consistent with previous studies that also found cultural differences in co-sleeping within the U.S comparing ethnic groups [67,68]. This implies cultural differences exists within the same region for sleeping arrangement.

Studies comparing infants and adolescents in the U.S. vs. infants in Japan [23] and adolescents in Italy [30] also found different associations between countries. Infants in the U.S. who were co-sleeping with their parents were more likely to have nocturnal night awakenings, bedtime protests and parent-reported stressful sleep disturbances. However, co-sleeping of infants in Japan only showed a significant association with nocturnal awakenings [23]. Furthermore bed/bedroom sharing was significantly associated with less success in maintaining and reinitiating sleep in adolescents in the U.S., however this was not found in Italy [30].

• School start/finish time.

School start and finish time was investigated in one study comparing adolescents in the U.S. vs. Australia [35]. Adolescents in the U.S. had a significantly earlier school start time than those in Australia, and school start time overall was found to have a significant effect on sleep duration. Results in this study found an average of 29 minutes less sleep for every hour that school start time was earlier; however, after controlling for the effect of living in either country to account for cultural differences, the significant impact of school start time on sleep duration was not maintained [35]. As the school start times were significantly different in each country, this finding supports the relationship that an earlier school start time may be associated with a shorter sleep duration. However, further research would explain this relationship in more detail, and be able to account for specific cultural variations which may also affect sleep duration. None of the studies investigated academic-related factors and their impact on sleep disturbances.

• Environment: Season and Daylight

Seasons and duration of daylight were compared in two studies [32,37]. One study compared season and daylight duration between school-aged children in Australia vs. Canada and found no significant differences [37]. Another study investigated the effect of season and daylight duration on nocturnal sleep duration of children across eight European countries. Although the study did not report the associations in each country, it was found that overall longer nocturnal sleep duration was significantly associated with fall/winter and shorter daylight duration [32]. However, after controlling for the effect of covariables (e.g., age, parental education level, country, overweight, screen time and time playing outside) no significant differences were found [32]. Therefore, the reported association between sleep duration and fall/winter, and daylight duration was likely due to the effect of culturally relevant factors in countries. Furthermore, no studies have investigated the impact of season and daylight on sleep disturbances.

## 4. Conclusions and Considerations for Future Research

To our knowledge, this is the first systematic review to explore studies that have investigated cultural differences in sleep duration and disturbances, and also cultural differences between child, parent and environmental factors and their association with sleep duration or disturbances, in a young population across two or more countries. The results of this review have demonstrated that there are specific sleep variables and culturally relevant factors that were prevalently studied in a particular age group, for example, habitual naps were mainly examined in those who are younger than pre-school-aged children while circadian rhythm was only examined in adolescents.

### 4.1. Cultural Considerations for Measuring Sleep

The studies utilised two main methods for assessing child sleep: parental/caregiver report and/or actigraphy. There are considerations for both of these methodologies when interpreting the findings and there may be benefits to using both. In Asia, parents reported more sleep disturbances. However, there is a high prevalence of bed/bedroom sharing with parents throughout Asia [17,42]. Therefore, this increase in parental report of sleep disturbances may be related to parental presence throughout the night, as they are more likely to identify sleep disturbances in their children with greater accuracy, compared to solitary sleeping practices [69]. In addition, the consideration of sleep throughout a 24 h period is important for contextualising nocturnal sleep findings. More frequent and longer habitual naps have been demonstrated in infants and children in Asia, and this is often embedded in a cultural belief that there are academic benefits to daytime napping in Asia [6]. Therefore, shorter nocturnal sleep of infants/toddlers and pre-school-aged children in Asia could be compensated by longer habitual naps during the day. The age when a child starts to habitually nap is a current controversy, and variability between countries in the timing of a monophasic sleep–wake pattern exists. For example, studies conducted in the U.S and Switzerland have reported that approximately 0-1% of 7-year-old children nap habitually [70,71]. A study in Korea reported that 5.6% of 7-year-old children had habitual naps, and it is increased further to 7.7% at 11 years old [72]. The methodology of the collection of data and the subsequent analysis is important to consider in future research. Previous studies found that parental reports of night awakenings were not associated with actigraphy measures of night awakenings [73] and parental reports on children’s sleep problems were overstated if parents had poor sleep quality [74]. Therefore, there is a justification for objective sleep measurements to be included in studies to examine children’s sleep disturbances accurately, without parental bias. In addition, our review has demonstrated the importance of gathering relevant demographic data to evaluate sleep, e.g., future studies should include co-sleeping and bed sharing to control for these factors.

### 4.2. Comparisons between Countries in Larger Regional Areas

Although our eligibility criteria incorporated studies that included two or more countries, many studies grouped countries by large regional areas in their analysis. These differences are problematic when synthesising the evidence. In addition, this review has demonstrated that this approach may not be sensitive in capturing cross-cultural differences found between individual countries within one region (e.g., countries within Asia [33,39,40] or within Europe [32]). These regional differences have been demonstrated in several studies, for example, one study compared pre-school-aged children in Japan with pre-school-aged children in China and found significant differences in bedtime, wake up time and total sleep duration [39]. Countries within the same region are more likely to share similar cultural values and beliefs; however, there might be other factors that contribute to significant differences in children’s sleep duration and disturbances, which relate directly to individual countries [33,39]. To example this, more night awakenings were shown in pre-school-aged children in China compared to children in Japan, which could be due to pre-school-aged children in China experiencing more sleep-disordered breathing (SDB) due to high rates of air pollution, than their peers in Japan [39,75]. Therefore, future studies should consider the potential diversity of each country within a region for comparisons of child sleep and associated cross-cultural factors.

### 4.3. The Consideration of Other Cultural Factors

As alluded to in Section 4.2, there are considerations for a number of factors that are important when considering sleep research. Many of these have not been studied in two or more countries and therefore are not included in our review findings. For example, religion is an important cultural factor in sleep research. Poor sleep quality of a nationwide sample for Presbyterian Church (USA) members was associated with religious doubts, after controlling for sociodemographic and behavioural factors (e.g., age, mental and physical health, stressful life events, etc.) [76]. Furthermore, a recent systematic review examining racial disparities in the sleep of preschool aged children within the U.S. found that white, non-Hispanic children were more likely to go to bed earlier, have more nocturnal sleep and nap less than most other racial and ethnic minorities [18]. Thus, racial variability must be considered even within one country. El-Sheikh et al. [77] observed that marital conflict of parents was associated with lower sleep duration, quality and higher daytime sleepiness, after controlling for child and sociodemographic factors (e.g., age, ethnicity, sex and socioeconomic status). Furthermore, several studies reported that children who were exposed to war-related or political conflict-related violence have frequent sleep disturbance such as refusal to go to sleep alone, frequent nocturnal awakenings and nightmares [78,79]. Future research is encouraged to explore further these key factors associated with sleep along with other influencing factors.

### 4.4. Identifying “True” Cultural Factors

Although most studies examined cross-cultural differences between child, parent and environmental factors and their association with sleep duration and disturbances, some studies just reported an overall association which cannot represent whether investigated factors are country specific factors [34,44,45]. A very limited number of studies (*n* = 2) identified the “true effect” of cultural factors on children’s sleep duration and disturbances. Meaning this association between factors and sleep duration or disturbances was attributed to the country the child was living in [32,35]. Therefore, more studies that control for these factors would be beneficial to show true differences between sleep duration and disturbances across countries.

### 4.5. Scope for Future Research

The current review provides a narrative overview of the literature; however, studies varied in age, nationality and methods, and therefore there are limited studies of which a meta-analysis could be conducted on (*n* = 7) [17,25,28,30,34,37,41]. Previous cross-cultural studies were limited to mostly neurotypical populations and only one cross-cultural study was found on children diagnosed with a neurodevelopmental condition (e.g., children with autism spectrum disorder) [41]. As insufficient sleep and sleep disturbances are highly reported in children with neurodevelopmental conditions, compared to typically developing peers, it is important to investigate cultural differences and sleep within this population [80,81]. In addition, it is currently unclear how cultural differences affecting sleep duration and disturbances may impact on children and adolescents’ cognitive development or academic performance. Finally, most of the research includes countries in Asia, Europe, Australasia and North America, and very few cross-cultural studies have been conducted in countries of Africa (*n* = 2) [26,28] and the Middle East region (*n* = 1) [38]. It is important to pilot existing sleep interventions to demonstrate effectiveness across multiple countries given cultural-specific adaptations that may need to be considered for families (e.g., bedtime practices, availability of rooms and the number of siblings). Finally, these findings have highlighted the importance of addressing children’s sleep internationally. Professionals in the field of paediatric sleep should develop country-specific sleep recommendations and address inadequate sleep duration and sleep disturbances by identifying and considering cultural factors that are likely to influence sleep duration and disturbances of children in specific countries.

## Figures and Tables

**Table 1:** Characteristics of included studies relating to sleep duration variables.

Authors (Year)	Included Countries	Ages (Mean)/Gender (% of Male Participants)	Sleep Measurement	Sleep Duration Variables Relevant to Review	Brief Summary of Findings
LeBourgeois et al. (2005) [30]	Italy (*n* = 776)	12–17 years (M = 14.6y) /55.4%	Adolescent Sleep Hygiene Scale	(1) Duration of habitual naps	Adolescents in Italy had significantly higher scores in a daytime sleep hygiene question (e.g., not having more than 1-h naps during the day) (M = 5.1) than adolescents in the U.S (M = 3.9) (*p* < 0.001)
	US (*n* = 572)	12–17 years (M = 14.6y) /41.2%			
Liu et al. (2005) [31]	US (*n* = 494)	4.83–11 years (M = 7.56 y) /51.2%	CSHQ	(1) Bedtime (2) Wake up time (3) Nocturnal sleep duration	Children in China had a significantly later bedtime (M = 9.03), earlier wake up time (M = 6.46), and shorter nocturnal sleep duration (M = 9.25 h) than children in the U.S (M_bedtime_ = 8.46, M_wakeuptime_ = 6.91, M_sleepduration_ = 10.15 h) (all *ps* < 0.001).
	China (*n* = 517)	7–13 years (M = 11.0 y) /47%			
Mindell et al. (2010) [17]	17 countries (*n* = 29,287) divided into		BISQ	(1) Bedtime (2) Wake up time (3) Nocturnal sleep duration (4) Number of habitual naps (5) Duration of habitual naps (6) Total sleep time	Significant differences in bedtime, wake up time, nocturnal sleep duration, number of habitual naps, duration of habitual naps and total sleep time were shown across 17 countries (All *ps* < 0.0001). Infants and toddlers in P-A countries had a significantly later bedtime (M = 21.44), later wake up time (M = 7.11), shorter nocturnal sleep duration (M = 9.19 h), more frequent habitual naps (M = 2.06), longer duration of habitual naps (M = 3.11 h) and shorter total sleep time (M = 12.31 h) than infants and toddlers in P-C countries (M_bedtime_ = 20.42, M_wakeuptime_ = 6.63, M_sleepduration_ = 10.0 h, M_numberofnaps_ = 2.04, M_durationofnaps_ = 3.01 h, M_totalsleeptime_ = 13.02 h) (all *ps* < 0.0001).
	P-C countries (*n* = 7960; AU, CA, NZ, US, UK) and	Birth–3 years (mean not reported) /52.5%			
	P-A countries (*n* = 21327; CN, HK, IN, ID, KR, JP, MY, PH, SG, TW, TH, VN).	Birth–3 years (mean not reported) /51.9%			
Hense et al. (2011) [32]	8 European countries (*n* = 31,543) including	2–9 years	A standardised 24-h recall	(1) Nocturnal sleep duration	Nocturnal sleep duration ranged from 9.5 h in Estonia to 11.2 h in Belgium. Children in Northern Europe (including Sweden, Germany and Belgium) had a significantly longer nocturnal sleep duration than children in Southern Europe (including Italy, Spain and Cyprus) and Eastern Europe (including Estonia and Hungary) (*p* < 0.001)
	Estonia (*n* = 1331)	(M = 5.8 y)/48.9%			
	Italy (*n* = 1643)	(M = 6.2 y)/52.0%			
	Hungary (*n* = 902)	(M = 6.7 y)/49.2%			
	Cyprus (*n* = 953)	(M = 6.3 y)/49.4%			
	Spain (*n* = 504)	(M = 5.5 y)/42.4%			
	Sweden (*n* = 1215)	(M = 5.7 y)/51.7%			
	Germany (*n* = 1586)	(M = 6.1 y)/51.5%			
	Belgium (*n* = 408)	(M = 5.5 y)/52.5%			
Kohyama et al. (2011) [33]	Japan (*n* = 872)	Birth–3 years (mean not reported) /48.6%	BISQ	(1) Bedtime (2) Wake up time (3) Nocturnal sleep duration (4) Number of habitual naps (5) Duration of habitual naps (6) Total sleep time	Infants and toddlers in Japan had significantly less frequent habitual naps (M = 1.44), and shorter duration of habitual naps (M = 2.19h), than infants and toddlers in other 11 Asian countries (M_numberofnaps_ = 2.09, M_durationofnaps_ = 3.15 h) (*p* < 0.001). There were no significant differences in bedtime (Japan, M = 21.29; 11 Asian countries, M = 21.45), wake up time (Japan, M = 7.14; 11 Asian countries, M = 7.11), nocturnal sleep duration (Japan, M = 9.42 h; 11 Asian countries, M = 9.18 h) and total sleep time (Japan, M = 11.62 h; 11 Asian countries, M = 12.33 h) between infants and toddlers in Japan vs. 11 other Asian countries (*p* > 0.001).
	11 Asian countries including CN, HK, IN, ID, KR, JP, MY, PH, SG, TW, TH and VN (*n* = 20,455)	Birth–3 years (mean not reported) /48.1%			
Dewald et al. (2012) [25]	Netherlands (*n* = 166)	12.2–16.5 years (M = 15.2 y) /28%	Sleep diaries, actigraphy	(1) Nocturnal sleep duration	The results of the sleep diary demonstrated that adolescents in the Netherlands had a significantly shorter nocturnal sleep duration (M = 7:44) than adolescents in Australia (M = 8:27) (*p* < 0.001). However, actigraphy results did not show a significant difference in nocturnal sleep duration between adolescents in the Netherlands (M = 8:04) and Australia (M = 8:03) (*p* > 0.05).
	Australia (*n* = 236)	13.3–18.9 years (M = 15.5 y) /65%			
Mindell et al. (2013) [34]	17 countries (*n* = 2590) divided into		BCSQ	(1) Bedtime (2) Wake up time (3) Number of habitual naps (4) Duration of habitual naps (5) Total sleep time	Significant differences in bedtime, wake up time, number of habitual naps, duration of habitual naps and total sleep time were shown across 14 countries (All *ps* < 0.0001). Children in P-A countries had a significantly later bedtime (M = 9.85), later wake up time (M = 7.62), more frequent habitual naps (M = 1.93), longer duration of habitual naps (M = 1.58 h) and a shorter total sleep time (M = 9.44 h) than infants and toddlers in P-C countries (M_bedtime_ = 8.15, M_wakeuptime_ = 7.12, M_numberofnaps_ = 1.32, M_durationofnaps_ = 0.52 h, M_totalsleeptime_ = 10.54 h) (All *ps* < 0.0001).
	P-C countries (*n* = 1139; AU, CA, NZ, US, UK)	3–6 years (mean not reported) /49.8%			
	P-A countries (*n* = 1447; CN, HK, IN, ID, KR, JP, MY, PH, SG, TW, TH, VN).	3–6 years (mean not reported) /50.2%			
Short et al. (2013) [35]	Australia (*n* = 385)	13–18 years (M = 15.57 y) /60%	Sleep diary	(1) Bedtime (2) Wake up time (3) Nocturnal sleep duration	Adolescents in the U.S. had a significantly earlier wake up time (M = 6:23) and shorter nocturnal sleep duration (M = 7 h 22 min) than adolescents in Australia (M_wakeuptime_ = 7:10, M_sleepduration_ = 8 h 17 min), all *ps* < 0.001. However, no significant difference was found in bedtime between adolescents in the U.S. (M = 22:28) and Australia (M = 22:36).
	US (*n* = 302)	13–19 years (M = 16.03 y) /35%			
Ahn et al. (2016) [36]	South Korea (*n* = 1036)	Birth–3 years (mean not reported) /50.8%	BISQ	(1) Bedtime (2) Nocturnal sleep duration (3) Number of habitual naps (4) Duration of habitual naps (5) Total sleep time	Significant differences in bedtime, nocturnal sleep duration, number of habitual naps, duration of habitual naps and total sleep time were shown across South Korea, P-A countries, and P-C countries (All *ps* < 0.001). Infants and toddlers in South Korea had the latest bedtime (M = 10:08 pm), the least frequent habitual naps (M = 1.64), the shortest duration of habitual naps (M = 2.47 h), and shortest total sleep time (M = 11.89 h) compared to infants and toddlers in P-A countries (M_bedtime_ = 9:25pm, M_numberofnaps_ = 2.08, M_durationofnaps_ = 3.15 h, M_totalsleeptime_ = 12.33 h) and P-C countries (M_bedtime_ = 8:25pm, M_numberofnaps_ = 2.04, M_durationofnaps_ = 3.01 h, M_totalsleeptime_ = 13.02 h). Nocturnal sleep duration of infants and toddlers in South Korea (M = 9.42 h) was significantly longer than nocturnal sleep duration of those in P-A countries (M = 9.12 h), but shorter than those in P-C countries (M = 10.01 h).
	P-C countries (*n* = 7960; AU, CA, NZ, US, UK) and	Birth–3 years (mean not reported) /52.5%			
	P-A countries (*n* = 21327; CN, HK, IN, ID, KR, JP, MY, PH, SG, TW, TH, VN) from Mindell et al. (2010) [17]	Birth–3 years (mean not reported) /51.9%			
Biggs et al. (2016) [37]	Australia (*n* = 87)	5–12 years (M = 9.6 y) /58%	Actigraphy, National Sleep Foundation 2004 Sleep in America questionnaire	(1) Bedtime (2) Wake up time (3) Nocturnal sleep duration	Actigraphy results demonstrated that children in Canada had a significantly later bedtime during weekdays (M = 21:13) and weekends (22:02), and an earlier wake up time at the weekend (M = 8:05), than children in Australia (M_bedtimeweekdays_ = 20:54, M_bedtimeweekends_ = 21:35, M_wakeuptimeweekends_ = 7:42) (all *ps* < 0.01). No significant differences were found for wake up time during weekdays or nocturnal sleep duration during the weekdays or weekends, between children in Canada (M_wakeuptimeweekdays_ = 8:41, M_nocturalsleepdurationweekdays_ = 493 min, M_nocturnalsleepdurationweekends_ = 485 min) and Australia (M_wakeuptimeweekdays_ = 8:31, M_nocturalsleepdurationweekdays_ = 491 min, M_nocturnalsleepdurationweekends_ = 480 min). Questionnaire-based nocturnal sleep results demonstrated significantly longer nocturnal sleep duration in children in Australia (M = 598 min) than children in Canada (M = 565 min) (*p* = 0.001).
	Canada (*n* = 101)	5–12 years (M = 9.0 y) /56%			
Mindell et al. (2017) [38]	ME countries (*n* = 669; Saudi Arabia, Egypt, Algeria, United Arab Emirates, Jordan, Morocco, Iraq, Kuwai, Oman, Palestinian territories, Libyan Arab Jamahiriya, Bahrain, Israel and 83 from other Arab countries)	Birth–3 years (mean not reported) /50.2%	BISQ	(1) Bedtime (2) Wake up time (3) Nocturnal sleep duration (4) Number of habitual naps (5) Duration of habitual naps (6) Total sleep time	Infants and toddlers in the Middle East had a significantly later bedtime (M = 10:45), later wake up time (M = 8:38), shorter nocturnal sleep duration (M = 9.15 h), and shorter total sleep time (M = 11.72 h) than infants and toddlers in P-A countries (M_bedtime_ = 9:26, M_wakeuptime_ = 7:06, M_nocturnalsleepduration_ = 9.19h, M_totalsleeptime_ = 12.31 h) and P-C countries (M_bedtime_ = 8:25, M_wakeuptime_ = 6:38, M_nocturnalsleepduration_ = 10.01 h, M_totalsleeptime_ = 13.02 h) (all *ps* < 0.001). For habitual naps, infants and toddlers in P-A countries had more frequent habitual naps (M = 2.06), and a longer duration of habitual naps (M = 3.11 h), followed by infants and toddlers in P-C countries (M_numberofhabitualnaps_ = 2.04, M_durationofhabitualnaps_ = 3.01 h) and in Middle East countries (M_numberofhabitualnaps_ = 1.90, M_durationofhabitualnaps_ = 2.57 h) (all *ps* < 0.001).
	P-C and P-A countries from Mindell et al. (2010; *n* = 29,287) [17]	Birth–3 years (mean not reported) /51.94%			
Takahashi et al. (2018) [39]	Japan (*n* = 505)	4–5 years (mean not reported) /47.3%	CSHQ	(1) Bedtime (2) Wake up time (3) Total sleep time	Children in China had a significantly later bedtime (M = 21.70), later wake up time (M = 7.66), and longer total sleep time (M = 9.54 h) than children in Japan (M_bedtime_ = 21.36, M_wakeuptime_ = 6.69, M_totalsleeptime_ = 9.03 h) (all *ps* < 0.001).
	China (*n* = 1909)	4–5 years (mean not reported) /53.0%			
Daban & Goh (2019) [40]	6 Southeast Asia countries (*n* = 5987) including	Birth–3 years	BISQ	(1) Bedtime (2) Wake up time (3) Nocturnal sleep duration (4) Number of habitual naps (5) Duration of habitual naps	Significant differences in bedtime, wake up time, nocturnal sleep duration, frequency of habitual naps and duration of habitual naps were found across 6 Southeast Asia countries (all *ps* < 0.0001).
	Indonesia (*n* = 967)	(mean not reported) /50.2%			
	Malaysia (*n* = 997)	(mean not reported) /50.4%			
	Philippines (*n* = 1034)	(mean not reported) /49.8%			
	Singapore (*n* = 1001)	(mean not reported) /51.6%			
	Thailand (*n* = 988)	(mean not reported) /49.2%			
	Vietnam (*n* = 1000)	(mean not reported) /49.4%			

Note. M: Mean; y: years; h: hours; min: minutes; Nocturnal sleep includes variables such as sleep duration at night and night-time sleep; Total sleep time indicates 24 h sleep including nap; BISQ: Brief Infant Sleep Questionnaire; BCSQ: Brief Child Sleep Questionnaire; CSHQ: Children’s Sleep Habits Questionnaire; P-C: Predominantly-Caucasian; P-A: Predominantly-Asian; ME: Middle East; AU: Australia; CA: Canada; NZ: New Zealand; US: United States; UK: United Kingdom; CN: China; HK: Hong Kong; IN: India; ID: Indonesia; KR: Korea; JP: Japan; MY: Malaysia; PH: Philippines; SG: Singapore; TW: Taiwan; TH: Thailand; VN: Vietnam.

**Table 2:** Characteristics of included studies relating to sleep disturbance variables.

Authors (Year)	Included Countries	Ages (Mean)/ Gender (% of Male Participants)	Sleep Measurement	Sleep Disturbance Variables Relevant to Review	Brief Summary of Findings
Latz et al. (1999) [23]	Japan (*n* = 56)	6–48 months (Mean not reported) /50.0%	Interview questions on sleep problems	(1) Frequency of night awakenings (2) Bedtime resistance (3) Sleep problems	Infants and toddlers in the U.S. were more likely to have more than 3 night awakenings (30%), more bedtime resistance (26%) and more stress-related sleep problems (25%) per week than infants and toddlers in Japan (20%, 20% and 13%, respectively) All *ps* < 0.05.
	US (*n* = 61)	6–48 months (Mean not reported) /46.0%			
LeBourgeois et al. (2005) [30]	Italy (*n* = 776)	12–17 years (M = 14.6 y) /55.4%	Adolescent Sleep–Wake Scale	(1) Frequency of night awakenings (2) Bedtime resistance (3) Sleep latency (4) Sleep quality	Adolescents in Italy had significantly better sleep quality than adolescents in the U.S with higher scores in night waking, bedtime resistance and sleep latency (i.e., falling asleep) dimension (all *ps* < 0.001; specific data were not reported).
	US (*n* = 572)	12–17 years (M = 14.6 y) /41.2%			
Liu et al. (2005) [31]	US (*n* = 494)	4.83–11 years (M = 7.56 y) /51.2%	CSHQ	(1) Frequency of night awakenings (2) Bedtime resistance (3) Sleep latency (4) Daytime sleepiness (5) Sleep problems	Children in China had significantly greater problems in night awakenings (M = 3.85), bedtime resistance (M = 7.92), daytime sleepiness (M = 10.22) and overall sleep problems (M = 42.11) than children in the U.S (M_nightwaking_ = 3.49; M_bedtimeresistance_ = 7.01; M_daytimesleepiness_ = 9.72; M_sleepproblem_ = 38.71). All *ps* < 0.05. No significant difference was found in sleep latency between children in China (M = 1.28) and children in the U.S (M = 1.26).
	China (*n* = 517)	7–13 years (M = 11.0 y) /47%				
Mindell et al. (2010) [17]	17 countries (*n* = 29,287) divided into		BISQ	(1) Frequency and duration of (2) night awakening (3) Bedtime resistance (4) Sleep latency (5) Sleep quality (6) Sleep problems	Significant differences in the frequency and duration of night awakenings, bedtime resistance, sleep latency, sleep quality and overall sleep problems were shown across 17 countries (All *ps* < 0.0001). Infants and toddlers in P-A countries had significantly greater problems in the frequency of night awakenings (M = 1.69), duration of night awakenings (M = 0.52), sleep latency (19.29%), sleep quality (56.4%) and overall sleep problems (51.9%) than infants and toddlers in P-C countries (M_frequencyofnightwakenings_ = 1.13; M_durationofnightawakenings_ = 0.41; M_sleeplatency_ = 9.57%; M_sleepquality_ = 73.6%; M_sleepproblem_ = 26.3%). However, no significant differences were found between infants and toddlers in P-A countries (M = 22.17%) and P-C countries (14.29%) in the percentage of children experiencing bedtime resistance.
	P-C countries (*n* = 7960; AU, CA, NZ, US, UK) and	Birth–3 years (mean not reported)/52.5%			
	P-A countries (*n* = 21327; CN, HK, IN, ID, KR, JP, MY, PH, SG, TW, TH, VN).	Birth–3 years (mean not reported)/51.9%			
Kohyama et al. (2011) [33]	Japan (*n* = 872)	Birth–3 years (mean not reported)/48.6%	BISQ	(1) Number and duration of (2) night awakening (3) Bedtime resistance (4) Sleep latency (5) Sleep problems	Infants and toddlers in Japan had significantly fewer night awakenings (M = 1.25) and shorter duration of night awakenings (M = 0.28 h) than infants and toddlers in 11 other Asian countries (M_frequencyofnightawakenings_ = 1.71; M_durationofnightawakenings_ = 0.53 h). Infants and toddlers in Japan were more likely to have bedtime resistance (M = 28.44%) and less likely to have sleep problems (M = 19.61%) than infants and toddlers in 11 other Asian countries (M_bedtimeresistance_ = 21.91%; M_sleepproblem_ = 53.28%). All *ps* < 0.001. No significant differences were found in the percentage of children having sleep latency >30 min between infants and toddlers in Japan (M = 22.71%) vs. in 11 other Asian countries (M = 19.15%).
	11 Asian countries including CN, HK, IN, ID, KR, JP, MY, PH, SG, TW, TH and VN (*n* = 20,455)	Birth–3 years (mean not reported)/48.1%			
Dewald et al. (2012) [25]	Netherlands (*n* = 166)	12.2–16.5 years (M = 15.2 y) /28%	Sleep diaries, actigraphy	(1) Sleep latency (2) Sleep quality	The actigraphy results showed no significant differences in sleep latency between adolescents in the Netherlands (M = 23 min) and in Australia (M = 23 min). The actigraphy results found significantly higher sleep quality in adolescents in the Netherlands (M = 80.04%) than peers in Australia (M = 77%) (*p* < 0.01), whereas sleep diary data found significantly higher sleep quality in adolescents in Australia (M = 96.66%) than peers in the Netherlands (M = 90.49%) (*p* < 0.001).
	Australia (*n* = 236)	13.3–18.9 years (M = 15.5 y) /65%			
Mindell et al. (2013) [34]	17 countries (*n* = 2590) divided into		BCSQ	(1) Frequency and duration of night awakening (2) Sleep latency (3) Daytime sleepiness (4) Sleep problems	Significant differences in the frequency of night awakenings, duration of night awakenings, sleep latency, daytime sleepiness and sleep problems were shown across 14 countries (All *ps* < 0.0001). Children in P-A countries had a significantly longer sleep latency (M = 21.25 min) and a greater percentage of sleep problems (M = 24.2%) than children in P-C countries (M_sleeplatency_ = 19.12mins; M_sleepproblem_ = 18.4%) (all *ps* < 0.0001). No significant differences were found in the frequency of night awakenings (M_P-C_ = 1.59; M_P-A_ = 1.66), duration of night awakenings (M_P-C_ = 7.19; M_P-A_ = 9.90) and daytime sleepiness (M_P-C_ = 12.6%; M_P-A_ = 9.5%) between children in P-C countries and P-A countries.
	P-C countries (*n* = 1139; AU, CA, NZ, US, UK)	3–6 years (mean not reported) /49.8%			
	P-A countries (*n* = 1447; CN, HK, IN, ID, KR, JP, MY, PH, SG, TW, TH, VN).	3–6 years (mean not reported) /50.2%			
Ahn et al. (2016) [36]	South Korea (*n* = 1036)	Birth–3 years (mean not reported) /50.8%	BISQ	(1) Frequency and duration of night awakenings	Infants and toddlers in South Korea had the shortest duration of nocturnal awakenings (M = 0.34 min) compared to infants and toddlers in P-A countries (M = 0.53 min) and in P-C countries (M = 0.41 min) (*p* < 0.001). Infants and toddlers in South Korea had less frequent night awakenings (M = 1.49) than infants and toddlers in P-A countries (M = 1.70), and more frequent night awakenings than infants and toddlers in P-C countries (M = 1.13) (*p* < 0.001).
	P-C countries (*n* = 7960; AU, CA, NZ, US, UK) and	Birth–3 years (mean not reported)/52.5%			
	P-A countries (*n* = 21327; CN, HK, IN, ID, KR, JP, MY, PH, SG, TW, TH, VN) from Mindell et al. (2010) [17]	Birth–3 years (mean not reported)/51.9%			
Biggs et al. (2016) [37]	Australia (*n* = 87)	5–12 years (M = 9.6 y) /58%	Actigraphy	(1) Duration of night awakening (2) Sleep latency (3) Sleep quality	Actigraphy results demonstrated no significant differences in the duration of night awakenings during weekdays (M_Australia_ = 77, M_Canada_ = 73), weekends (M_Australia_ = 76, M_Canada_ = 70), sleep latency during weekdays (M_Australia_ = 28 min, M_Canada_ = 27mins) or weekends (M_Australia_ = 26 min, M_Canada_ = 20 min), and sleep quality during weekdays (M_Australia_ = 79%, M_Canada_ = 81%) or weekends (M_Australia_ = 79%, M_Canada_ = 81%) between children in Australia vs. children in Canada (All *ps* > 0.05).
	Canada (*n* = 101)	5–12 years (M = 9.0 y) /56%			
Irwanto et al. (2016) [41]	Indonesia (*n* = 25)	4–10 years of children with ASD (M = 4.8 y) /84%	CSHQ-A	(1) Bedtime resistance (2) Frequency of night awakenings (3) Sleep problems	Children with ASD in Indonesia had a significantly greater frequency of night awakenings (M = 2.76) and overall sleep problems (M = 41.12) than children with ASD in Japan (M_nightwaking_ = 2.36; M_sleepproblem_ = 37.28) (all *ps* < 0.05). There was no significant difference in bedtime-related sleep problems between children with ASD in Indonesia (M = 18.88) and children with ASD in Japan (M = 17.88; *p* > 0.05).
	Japan (*n* = 25)	4–10 years of children with ASD (M = 7.7 y) /80%			
Mindell et al. (2017) [38]	ME countries (*n* = 669; Saudi Arabia, Egypt, Algeria, United Arab Emirates, Jordan, Morocco, Iraq, Kuwai, Oman, Palestinian territories, Libyan Arab Jamahiriya, Bahrain, Israel and 83 from other Arab countries)	Birth–3 years (mean not reported) /50.2%	BISQ	(1) Frequency and duration of night awakenings (2) Bedtime resistance (3) Sleep problem	Infants and toddlers in the Middle East had a significantly higher frequency of night awakenings (M = 2,20), a longer duration of night awakenings (M = 41 min) and a greater percentage of bedtime resistance (38%), compared with infants and toddlers in P-A countries (M_frequencyrofnightawakenings_ = 1.69; M_durationofnightawakenings_ = 31mins; M_bedtimeresistance_ = 22.2%) and P-C countries (M_frequencyrofnightawakenings_ = 1.13; M_durationofnightawakenings_ = 25 min; M_bedtimeresistance_ = 14.3%). All *ps* < 0.001. Infants and toddlers in P-A countries had the greatest percentage of sleep problems (51.9%), compared with toddlers in Middle East countries (36.9%) and in P-C countries (26.3%) (*p* < 0.001).
	P-C and P-A countries from Mindell et al. (2010; *n* = 29,287) [17]	Birth–3 years (mean not reported) /51.94%			
Takahashi et al. (2018) [39]	Japan (*n* = 505)	4–5 years (mean not reported) /47.3%	CSHQ	(1) Frequency of night awakenings (2) Bedtime resistance (3) Sleep latency (4) Daytime sleepiness (5) Sleep problems	Children in China had significantly greater sleep problems in the frequency of night awakenings (M = 4.09) and sleep latency (M = 1.61) than children in Japan (M_frequencyofnightawakening_ = 3.47; M_sleeplatency_ = 1.43), whereas children in China had significantly less problems in bedtime resistance (M = 11.05) than children in Japan (M = 12.49). All *ps* < 0.05. No significant differences between countries were found in daytime sleepiness (M_Japan_ = 10.76; M_China_ = 11.12) and overall sleep problems (M_Japan_ = 46.56; M_China_ = 47.35).
	China (*n* = 1909)	4–5 years (mean not reported) /53.0%			
Daban & Goh (2019) [40]	6 Southeast Asia countries (*n* = 5987) including	Birth–3 years	BISQ	(1) Frequency and duration of night awakenings (2) Sleep latency (3) Sleep problem	Significant differences were found in the frequency of night awakenings, the duration of night awakenings, sleep latency and sleep problems across 6 Southeast Asia countries (all *ps* > 0.0001).
	Indonesia (*n* = 967)	(mean not reported) /50.2%				
	Malaysia (*n* = 997)	(mean not reported) /50.4%			
	Philippines (*n* = 1034)	(mean not reported) /49.8%			
	Singapore (*n* = 1001)	(mean not reported) /51.6%			
	Thailand (*n* = 988)	(mean not reported) /49.2%			
	Vietnam (*n* = 1000)	(mean not reported) /49.4%			
van Selms et al. (2019) [29]	Netherland (*n* = 1131)	7–12 years (M = 10.0) /44.6%	Simple author questions	(1) Sleep latency	A significantly greater percentage of children in Netherland had sleep latency (i.e., difficulty falling asleep; 60.4%), than children in Armenia (30.8%) and children in Indonesia (21.5%) (*p* < 0.01).
	Armenia (*n* = 886)	7–12 years (M = 9.1) /49.0%			
	Indonesia (*n* = 545)	7–12 years (M = 9.5) /41.5%			

Note. M: Mean; y: years; h: hours; min: minutes; BISQ: Brief Infant Sleep Questionnaire; BCSQ: Brief Child Sleep Questionnaire; CSHQ: Children’s Sleep Habits Questionnaire; P-C: Predominantly-Caucasian; P-A: Predominantly-Asian; ME: Middle East; AU: Australia; CA: Canada; NZ: New Zealand; US: United States; UK: United Kingdom; CN: China; HK: Hong Kong; IN: India; ID: Indonesia; KR: Korea; JP: Japan; MY: Malaysia; PH: Philippines; SG: Singapore; TW: Taiwan; TH: Thailand; VN: Vietnam; ASD: Autism Spectrum Disorder: CSHQ-A: Children’s Sleep Habits Questionnaire-Abbreviated.

**Table 3:** Cultural factors examined with sleep duration or sleep disturbances.

Authors (Year)	Included Countries	Ages (Mean)/ Gender (% of Male Participants)	Cultural Factor Domains		
			Child	Parental	Environmental
Latz et al. (1999) [23]	Japan (*n* = 56)	6–47 months (M = 30.3) /72%	not measured	Parental education level, Sleep initiating and resuming methods	Number in household, Maternal employment, Co-sleeping in body contact, Adult company at bedtime
	US (*n* = 61)	6–47 months (M = 25.0) /67%			
LeBourgeois et al. (2005) [30]	Italy (*n* = 776)	12–17 years (M = 14.6 y) /55.4%	Physiologically sleep-inhibiting, cognitive and emotional status at bedtime, Circadian preference, Puberty	Bedtime routine	SES, Bed/bedroom sharing
	US (*n* = 572)	12 to 17 years (M = 14.6 y) /41.2%			
Mindell et al. (2010) [17]	17 countries (*n* = 29,287) divided into		not measured	Maternal education level, Bedtime routine	Sleeping location, Maternal employment
	P-C countries (*n* = 7960; AU, CA, NZ, US, UK) and	Birth–3 years (mean not reported)/52.5%			
	P-A countries (*n* = 21327; CN, HK, IN, ID, KR, JP, MY, PH, SG, TW, TH, VN).	Birth–3 years (mean not reported)/51.9%			
Mindell et al. (2010) [42]	17 countries (*n* = 29,287) divided into P-C countries (*n* = 7960; AU, CA, NZ, US, UK) and P-A countries (*n* = 21327; CN, HK, IN, ID, KR, JP, MY, PH, SG, TW, TH, VN).	Birth–3 years (mean not reported)/51.9% (for entire sample)	not measured	Sleep initiating and resuming method	Sleeping location
Hense et al. (2011) [32]	8 European countries (*n* = 31,543) including	2–9 years	Overweight, Playing outdoors, Time spent in front of TV or PC	Parental education level	Daylight, season
	Estonia (*n* = 1331)	(M = 5.8 y)/48.9%			
	Italy (*n* = 1643)	(M = 6.2 y)/52.0%			
	Hungary (*n* = 902)	(M = 6.7 y)/49.2%			
	Cyprus (*n* = 953)	(M = 6.3 y)/49.4%			
	Spain (*n* = 504)	(M = 5.5 y)/42.4%			
	Sweden (*n* = 1215)	(M = 5.7 y)/51.7%			
	Germany (*n* = 1586)	(M = 6.1 y)/51.5%			
	Belgium (*n* = 408)	(M = 5.5 y)/52.5%			
Kohyama et al. (2011) [33]	Japan (*n* = 872)	Birth–3 years (mean not reported)/48.6%	not measured	Bedtime routine	Sleeping location, Parental presence at bedtime
	11 Asian countries including CN, HK, IN, ID, KR, JP, MY, PH, SG, TW, TH and VN (*n* = 20,455)	Birth–3 years (mean not reported)/48.1%			
Sadeh et al. (2011) [24]	17 countries (*n* = 29,287) divided into P-C countries (*n* = 7960; AU, CA, NZ, US, UK) and P-A countries (*n* = 21327; CN, HK, IN, ID, KR, JP, MY, PH, SG, TW, TH, VN).	Birth–3 years (mean not reported)/48.1% (for entire sample)	not measured	Maternal education level	Presence of siblings, Maternal employment
Mindell et al. (2013) [34]	17 countries (*n* = 2590) divided into		Daily outdoor time, Screen time (i.e., television viewing, using a computer, playing other electronic games)	Bedtime routine, Maternal education level	Maternal employment, Sleeping location
	P-C countries (*n* = 1139; AU, CA, NZ, US, UK)	3–6 years (mean not reported) /49.8%			
	P-A countries (*n* = 1447; CN, HK, IN, ID, KR, JP, MY, PH, SG, TW, TH, VN).	3–6 years (mean not reported) /50.2%			
Short et al. (2013) [35]	Australia (*n* = 385)	13–18 years (M = 15.57 y) /60%	School start time, Extracurricular load, Circadian preference	Parent-set bedtime	not measured
	US (*n* = 302)	13–19 years (M = 16.03 y) /35%			
Chaput et al. (2015) [43]	12 countries (*n* = 5777) including	9–11 years	Diet, Physical activity, Screen time (i.e., hours of watching TV, playing video games and using computer)	not measured	not measured
	Australia (*n* = 433)	(M = 10.7 y)/46.7%			
	Canada (*n* = 496)	(M = 10.5 y)/40.9%			
	China (*n* = 459)	(M = 9.9 y)/51.6%			
	India (*n* = 433)	(M = 10.5 y)/45.1%			
	UK (*n* = 374)	(M = 10.9 y)/42.8%			
	US (*n* = 421)	(M = 9.9 y)/40.4%			
	Brazil (*n* = 435)	(M = 10.5 y)/48.5%			
	Colombia (*n* = 820)	(M = 10.5 y)/49.2%			
	Finland (*n* = 526)	(M = 10.4 y)/45.3%			
	Kenya (*n* = 452)	(M = 10.2 y)/45.4%			
	Portugal (*n* = 563)	(M = 10.4 y)/41.6%			
	South Africa (*n* = 452)	(M = 10.2 y)/38.6%			
Mindell et al. (2015) [44]	13 countries including AU/NZ, CA, UK, US, CN, HK, IN, JP, KR, MY, PH, SG and TH (*n* = 10085)	Birth–5.11 years (mean not reported)/49.6% (for entire sample)	not measured	Bedtime routine	not measured
Mindell et al. (2015) [45]	13 countries (*n* = 10085) including		not measured	Maternal education level, Maternal sleep	Number of children, Maternal employment
	P-C countries (*n* = 4152; AU/NZ, CA, UK, US) and	Birth–6 years (mean not reported)/ 50.7%			
	P-A countries (*n* = 5933; CN, HK, IN, JP, KR, MY, PH, SG and TH)	Birth–6 years (mean not reported)/ 50.2%			
Vazsonyi et al. (2015) [26]	Swiss (*n* = 5575)	15–18 years (M = 17.17)/ 50.0%	not measured	Parental warmth	not measured
	Georgia (*n* = 6692)	15–18 years (M = 15.83)/ 40.0%			
Biggs et al. (2016) [37]	Australia (*n* = 87)	5–12 years (M = 9.6 y) /58%	not measured	Maternal education level	Season, Daylight amount
	Canada (*n* = 101)	5–12 years (M = 9.0 y) /56%			
Mindell et al. (2017) [38]	ME countries (*n* = 669; Saudi Arabia, Egypt, Algeria, United Arab Emirates, Jordan, Morocco, Iraq, Kuwai, Oman, Palestinian territories, Libyan Arab Jamahiriya, Bahrain, Israel and 83 from other Arab countries)	Birth–3 years (mean not reported) /50.2%	not measured	Bedtime routine	not measured
	P-C and P-A countries from Mindell et al. (2010; *n* = 29,287) [17]	Birth–3 years (mean not reported) /51.94%			
Takahashi et al. (2018) [39]	Japan (*n* = 505)	4–5 years (mean not reported) /47.3%	not measured	Parental education level	Family structure, Number of siblings
	China (*n* = 1909)	4–5 years (mean not reported) /53.0%			
Carneiro et al. (2019) [28]	Cape Verde (*n* = 206)	2–15 years (mean not reported) /46%	Bedtime television	Parental education level	Number of cohabitants, Number of cohabitant children
	Mozambique (*n* = 438)	4–13 years (mean not reported) /49%			
Daban & Goh (2019) [40]	6 Southeast Asia countries (*n* = 5987) including	Birth–3 years	not measured	Bedtime routine	Sleeping location
	Indonesia (*n* = 967)	(mean not reported) /50.2%			
	Malaysia (*n* = 997)	(mean not reported) /50.4%			
	Philippines (*n* = 1034)	(mean not reported) /49.8%			
	Singapore (*n* = 1001)	(mean not reported) /51.6%			
	Thailand (*n* = 988)	(mean not reported) /49.2%			
	Vietnam (*n* = 1000)	(mean not reported) /49.4%			
van Selms et al. (2019) [29]	Netherland (*n* = 1131)	7–12 years (M = 10.0) /44.6%	Pressure from home, Pressure from school, Easily scared, Worried	not measured	not measured
	Armenia (*n* = 886)	7–12 years (M = 9.1) /49.0%			
	Indonesia (*n* = 545)	7–12 years (M = 9.5) /41.5%			
			7	17	13

Note. M: Mean; y: years; P-C: Predominantly-Caucasian; P-A: Predominantly-Asian; ME: Middle East; AU: Australia; CA: Canada; NZ: New Zealand; US: United States; UK: United Kingdom; CN: China; HK: Hong Kong; IN: India; ID: Indonesia; KR: Korea; JP: Japan; MY: Malaysia; PH: Philippines; SG: Singapore; TW: Taiwan; TH: Thailand; VN: Vietnam, SES: Socio-Economic Status.

**Table 4:** Universal or cultural factors associated with sleep duration or disturbances in young populations.

	Universal Factors	Cultural Factors
**Child factors**		
Physical health-related factors	Physiological status at bedtime [30] Diet [43]	
Psychological health-related factors	Emotional status [30] Cognitive status [30] Pressure from school [29] Worried [29]	Pressure from home [29] Easily scared [29]
Physical activities		Vigorous physical activity [43]
Screen time		Screen time [28,43]
Academic related factors	NA	NA
Adolescent-related factors	Circadian preference [30] Puberty status [30]	
**Parent factors**		
Parenting practices	Parental warmth [26] Bedtime routine [30]	
Parent-related factor		Maternal education level [24,28] Paternal education level [28]
**Environmental factors**		
Family composition	The number of families [28] The number of siblings [28]	Presence of siblings [24]
Lifestyles	SES [30]	Maternal employment status [24]
Sleeping arrangement		Bed/bedroom sharing [30] Falling asleep alone [42] Co-sleeping with body contact [23]

Note. Findings in this table included studies that examined overall associations between factors and sleep duration or disturbances [34,38,44,45]; NA: Not applicable.

## Appendix A

**Table A1:** Table summarising the results of the CASP case–control critical appraisal of all case–control study design papers.

Criterion	Latz et al. [23]	Iwawaki & Schuller [16]	LeBourgeois et al. [30]	Liu et al. [31]	Mindell et al. [17]	Mindell et al. [42]	Hense et al. [32]	Kohyama et al. [33]	Sadeh et al. [24]	Dewald et al. [25]	Mindell et al. [34]	Short et al. [35]	Varzsonyl et al. [26]	Chaput et al. [43]	Mindell et al. [44]	Mindell et al. [45]	Ahn et al. [36]	Biggs et al. [37]	Irwanto et al. [41]	Tynjälä et al. [27]	Mindell et al. [38]	Takahashi et al. [39]	Carneiro et al. [28]	Daban & Goh [40]	van Selms et al. [29]
Clearly focused question	✓	✓	✓	✓	✓	✓	✓	✓	✓	✓	✓	✓	✓	✓	✓	✓	✓	✓	✓	✓	✓	✓	✓	✓	✓
Appropriate design	✓	✓	✓	✓	✓	✓	✓	✓	✓	✓	✓	✓	✓	✓	✓	✓	✓	✓	✓	✓	✓	✓	✓	✓	✓
Clearly defined recruitment	✓	✓	✓	✓	✓	✓	✓	✓	✓	✓	✓	✓	✓	✓	✓	✓	✓	✓	✓	✓	✓	✓	✓	✓	✓
Sample size based on a power calculation ^a^	n/a	n/a	n/a	n/a	n/a	n/a	n/a	n/a	n/a	n/a	n/a	n/a	n/a	n/a	n/a	n/a	n/a	n/a	n/a	n/a	n/a	n/a	n/a	n/a	n/a
Outcome measurements valid and reliable	✕	✕	✓	✓	✓	✓	✓	✓	✓	✓	✓	✓	✕	✓	✓	✓	✓	✓	✓	✕	✓	✓	✓	✓	✕
Exposure measurements valid and reliable	✕	✓	✓	n/a	p	p	p	p	p	n/a	p	✕	✕	p	p	✓	p	✕	n/a	✕	✓	✕	✕	✓	✕
Confounding factors accounted	✕	✕	✓	✓	✓	✓	✓	✕	✕	✕	✓	✓	✓	✓	✓	✓	✕	✓	✕	✕	✕	✓	✕	✕	✕
Appropriate statistical analysis	✓	✕	✓	✓	✓	✓	✓	✓	✓	✓	✓	✓	✓	✓	✓	✓	✓	✓	✓	✕	✓	✓	✓	✓	✓
Precise estimate of effect	✕	✓	✕	✓	✓	✓	✓	✓	✕	✓	✓	✓	✓	✓	✓	✓	✓	✓	✕	✕	✓	✓	✕	✓	✓
Reliable results with acknowledged possible bias	✓	✕	✓	✓	✓	✓	✓	✓	✓	✓	✓	✓	✓	✓	✓	✓	✓	✓	✓	✕	✓	✓	✓	✓	✓
Ability to generalize results	✕	✕	✕	✕	✕	✕	✓	✕	✕	✕	✕	✕	✕	✕	✕	✕	✕	✕	✕	✕	✕	✓	✕	✕	✕
Interpretation related to the existing evidence	✓	✕	✓	✓	✓	✓	✓	✓	✓	✓	✓	✓	✓	✓	✓	✓	✓	✓	✓	✓	✓	✓	✓	✓	✓
**Overall Methodological Quality**	**L**	**L**	**M**	**M**	**M**	**M**	**H**	**M**	**L**	**L**	**M**	**M**	**L**	**M**	**M**	**M**	**M**	**M**	**M**	**L**	**M**	**M**	**L**	**M**	**L**

Note: ✓—Yes; ✕—No; n/a—not applicable (^a^ it means the study did not mention a power calculation); p—partially; L—Low (≤8); M—Moderate (≤10); H—High (≤12).

## Appendix B

**Table A2:** Cultural factors examined with sleep duration or sleep disturbances.

Authors (Year)	Included Countries	Ages (Mean)/ Gender (% of Male Participants)	Cultural Factor Domains			Detailed Results
			Child	Parent	Environmental	
Latz et al. (1999) [23]	Japan (*n* = 56)	6–47 months (M = 30.3)/ 72%	not measured	Parental education level, sleep initiating and resuming methods	Number in household, maternal employment, co-sleeping in body contact, adult company at bedtime	***Parental*** *Differences in parental factors between countries* **- Mother’s education (years)**: American (14.2) < Japanese (14.6) **- Father’s education (years)**: American (14.6) < Japanese (16.0) *** **- Sleep initiating and resuming methods**: **Bottle in bed**: Japanese (9%) < American (20%) **Body contact when children woke at night**: Japanese (9%) < American (61%) ** ***Environmental*** *Differences in environmental factors between countries* **- No. in household**: American (4.1) < Japanese (4.2) **- Mother employed outside home**: Japanese (20%) < American (26%) **- Co-sleeping (in body contact, ≥3 a week)**: American (15%) < Japanese (59%) *** **- Adult company at bedtime**: American (16%) < Japanese (68%) *** *Associations between co-sleeping and sleep disturbances in each country* **US**: Co-sleeping with body contact was associated with more bedtime protests,*** night awakenings** and overall stressful sleep problems.** **Japan**: Co-sleeping with body contact was only associated with increased night awakenings.*
	US (*n* = 61)	6–47 months (M = 25.0)/ 67%				
LeBourgeois et al. (2005) [30]	Italy (*n* = 776)	12–17 years (M = 14.6y) /55.4%	Physiologically sleep-inhibiting, cognitive and emotional status at bedtime, circadian preference, puberty	Bedtime routine	SES, Bed/bedroom sharing	***Child*** *Differences in child factors between countries* **- Physiological status (Score)**: American (3.9) < Italian (4.7) *** **- Cognitive status (score)**: American (3.3) < Italian (4.0) *** **- Emotional status (score)**: American (4.2) < Italian (4.7) *** **- Circadian preference (Morning/Eveningness scale score)**: American (26.0) < Italian (26.3) **- Pubertal status (Pubertal Developmental Scale score)**: Italian (3.1) < American (3.2) *Associations with sleep disturbances in each country* **- Physiologically sleep-inhibiting status** was not significantly associated with the sleep quality of adolescents in the U.S. and Italy. **- Better cognitive status** had a significantly positive association with sleep quality of adolescents in the U.S. and Italy.*** **- Better emotional status** had a significantly positive association with sleep quality of adolescents in the U.S. and Italy.*** **- Morningness** was associated with significantly better overall sleep quality than eveningness in adolescents in the U.S. and Italy.*** **- Pubertal status** was not significantly associated with the sleep quality of adolescents in the U.S. and Italy ***Parental*** *Differences in parental factors between countries* **Bedtime routine (score)**: American (3.9) < Italian (4.1) *Associations between bedtime routine and sleep disturbances in each country* **Bedtime routine** was not associated with the sleep quality of adolescents in the U.S. and Italy. ***Environmental*** *Differences in environmental factors between countries* **- SES (head of household’s occupation on a scale of 1 (unskilled) to 9 (professional))**: Italian (4.5) < American (5.1) *** **- No bed/bedroom sharing (score)**: Italian (4.5) < American (5.1) *** *Associations with sleep disturbances in each country* **- SES** was not significantly associated with sleep quality in both adolescents in the U.S. and Italy. **- Bed/Bedroom sharing** **US**: Bed/bedroom sharing was significantly associated with less success in maintaining and reinitiating sleep.*** **Italy**: Significant association was not shown.
	US (*n* = 572)	12 to 17 years (M = 14.6y) /41.2%				
Mindell et al. (2010) [17]	17 countries (*n* = 29,287) divided into		not measured	Maternal education level, bedtime routine	Sleeping location, maternal employment	***Parental*** *Differences in parental factors between countries* **- Maternal education level** *** Elementary school (%): P-A (1.05) < P-C (1.43) High school (%): P-A (39.31) < P-C (42.45) College (%): P-C (38.15) < P-A (42.76) Postgraduate (%): P-A (16.88) < P-C (17.97) **- Consistent bedtime routine (%)**: P-C (60.65) < P-A (71.13) **** ***Environmental*** *Differences in environmental factors between countries* **- Sleeping Location** **Sleep in parent’s bed**: P-C (11.8%) < P-A (64.65%) **** **Sleep in parent’s room**: P-C (21.95%) < P-A (86.47%) **** **Sleep in own room**: P-A (7.03%) < P-C (62.54%) **** **- Mother’s employment status** *** Full time (%): P-C (29.86) < P-A (60.41) Part time (%): P-A (4.86) < P-C (15.36) Home/student (%): P-A (34.73) < P-C (54.7) *Controlling sleep location examining differences in sleep duration and disturbances between countries* - Significant differences in sleep duration and disturbances between P-C and P-A countries were maintained, except for the longest period of consolidated sleep.***
	P-C countries (*n* = 7960; AU, CA, NZ, US, UK) and	Birth–3 years (mean not reported)/52.5%				
	P-A countries (*n* = 21327; CN, HK, IN, ID, KR, JP, MY, PH, SG, TW, TH, VN).	Birth–3 years (mean not reported)/51.9%				
Mindell et al. (2010) [42]	17 countries (*n* = 29,287) divided into P-C countries (*n* = 7960; AU, CA, NZ, US, UK) and P-A countries (*n* = 21327; CN, HK, IN, ID, KR, JP, MY, PH, SG, TW, TH, VN).	Birth–3 years (mean not reported)/51.9% (for entire sample)	not measured	Sleep initiating and resuming method	Sleeping location	***Parental*** *Differences in parental factors between countries* ***- Sleep initiation methods*** **Bottle-feeding (%)**: P-C (16.01) < P-A (37.25) **** **Nursing (%)**: P-C (19.74) < P-A (28.07) **** **Rocking (%)**: P-C (20.69) < P-A (23.50) **** **Holding (%)**: P-A (26.11) < P-C (26.63) **Watching TV (%)**: P-C (4.41) < P-A (6.85) **- Sleep resuming methods** **Holding or rocking to sleep (%)**: P-C (21.09) < P-A (28.77) **** **Picking up—returning awake (%)**: P-A (5.18) < P-C (18.47) **** **Rub or pat in crib/bed (%)**: P-C (28.51) < P-A (31.89) **** **Giving a bottle (%)**: P-C (21.07) < P-A (33.61) **** **Nurse back to sleep (%)**: P-C (25.00) < P-A (29.30) **** **Verbal comfort in crib (%)**: P-A (13.06) < P-C (15.95) **** **Bring child to parents’ bed (%)**: P-A (7.46) < P-C (19.37) **** **Let cry to fall asleep (%)**: P-A (1.97) < P-C (15.65) **** **Wait a few minutes (%)**: P-A (21.30) < P-C (45.53) **** **Play until ready for sleep (%)**: P-C (1.22) < P-A (5.58) **** **Watch TV or video (%)**: P-A (1.03) < P-C (1.39) **Sing to child (%)**: P-C (8.74) < P-A (11.98) **** ***Environmental*** *Differences in environmental factors between countries* **- Sleeping location** **In crib/bed alone in the room**: P-A (3.58%) < P-C (56.98%) **** **In parent’s bed alone**: P-C (2.00%) < P-A (5.50%) **** **In crib/bed with parent present**: P-A (11.91%) < P-C (12.45%) **In parents’ bed with parent**: P-C (10.99%) < P-A (38.41%) **** **In another room of the house**: P-A (2.30%) < P-C (5.01%) **** *Associations between falling asleep alone and, sleep duration and disturbances in each country* **P-C**: falling asleep alone in own crib/bed was a predictor for longer nocturnal sleep, fewer night awakenings and longest continuous sleep episode. **P-A**: Sleep duration and disturbances were not predicted.
Hense et al. (2011) [32]	8 European countries (*n* = 31,543) including	2–9 years	Overweight, playing outdoors, time spent in front of TV or PC	Parental education level	Daylight, season	***Child*** *Associations with sleep duration after controlling for other cultural factors* **- Overweight**: Overweight had a significantly positive association with sleep duration,*** but the association was disappeared after controlling for other factors (e.g., age, parental education level, country, season, daylight hours, screen time and time playing outside). **- Playing outdoors**: Playing outdoors had a significantly positive association with sleep duration,*** but the association was disappeared after controlling for other factors (e.g., age, parental education level, country, season, daylight hours, overweight and screen time). **- Screen time (Time spent in front of TV or PC)**: Screen time had a significantly negative association with sleep duration,*** but the association was disappeared after controlling for other factors (e.g., age, parental education level, country, season, daylight hours, overweight and time playing outside). ***Parental*** *Associations with sleep duration after controlling for other cultural factors* **- Parental education level**: Children with medium or low parental education level were more likely to have shorter nocturnal sleep duration than those with high parental education level.*** However, the association was disappeared when other factors were controlled (e.g., age, country, season and daylight hours) ***Environmental*** *Associations with sleep duration after controlling for other cultural factors* **- Daylight**: Longer daylight hours was significantly associated with shorter duration of sleep.*** However, the association was disappeared when other factors were controlled (e.g., age, parental education level, country, seasons, overweight, screen time and time playing outdoors). **- Season**: Fall/winter was significantly associated with longer duration of sleep than spring/summer*** However, the association was disappeared when other factors were controlled (e.g., age, parental education level, country and daylight hours).
	Estonia (*n* = 1331)	(M = 5.8 y)/48.9%				
	Italy (*n* = 1643)	(M = 6.2 y)/52.0%				
	Hungary (*n* = 902)	(M = 6.7 y)/49.2%				
	Cyprus (*n* = 953)	(M = 6.3 y)/49.4%				
	Spain (*n* = 504)	(M = 5.5 y)/42.4%				
	Sweden (*n* = 1215)	(M = 5.7 y)/51.7%				
	Germany (*n* = 1586)	(M = 6.1 y)/51.5%				
	Belgium (*n* = 408)	(M = 5.5 y)/52.5%				
Kohyama et al. (2011) [33]	Japan (*n* = 872)	Birth–3 years (mean not reported)/48.6%	not measured	Bedtime routine	Sleeping location, parental presence at bedtime	***Parental*** *Differences in parental factors between countries* **- Consistent bedtime routine (%)**: Other Asian countries (60.13) < Japan (72.71).*** ***Environmental*** *Differences in environmental factors between countries* **- Sleeping location** **Parent’s bed**: Other Asian countries (64.43%) < Japan (69.72%) **Parent’s room**: Other Asian countries (86.40%) < Japan (88.07%) **Own room**: Japan (2.98%) < Other Asian countries (7.20%) *** **Parental presence at bedtime**: Japan (97.80%) < Other Asian countries (99.56%) ***
	11 Asian countries including CN, HK, IN, ID, KR, JP, MY, PH, SG, TW, TH and VN (*n* = 20,455)	Birth–3 years (mean not reported)/48.1%				
Sadeh et al. (2011) [24]	17 countries (*n* = 29,287) divided into P-C countries (*n* = 7960; AU, CA, NZ, US, UK) and P-A countries (*n* = 21327; CN, HK, IN, ID, KR, JP, MY, PH, SG, TW, TH, VN).	Birth–3 years (mean not reported)/48.1% (for entire sample)	not measured	Maternal education level	Presence of siblings, maternal employment	***Parental*** *The impact of maternal education level on sleep disturbances in each country* **P-A: Maternal education level** had a significant effect on the parental definition of “severe sleep problems”.**** Parents with lower education level were less likely to define their child’s sleep as a severe sleep problem. **P-C**: Significant effect of maternal education level was not shown. ***Environmental*** *The impact of environmental factors on sleep disturbances in each country* **- Maternal employment** **P-A**: Mother’s employment had a significant effect on the parental definition of “severe sleep problems”.**** Mothers with higher employment level were more likely to define their child’s sleep as a severe sleep problem. **P-C**: Significant effect of maternal employment was not shown. **- Presence of siblings (only child vs. other children)** **P-A**: Presence of siblings had a significant effect on the parental definition of “severe sleep problems”.**** This indicated that only child’s sleep was less likely to be defined as a severe sleep problem than those with siblings. **P-C**: Significant effect of the presence of siblings was not shown.
Mindell et al. (2013) [34]	17 countries (*n* = 2590) divided into		Daily outdoor time, Screen time (i.e., television viewing, using a computer, playing other electronic games)	Bedtime routine, maternal education level	Maternal employment, sleeping location	***Child*** *Differences in child factors between countries* **- Daily outdoor time**: P-C (2.30 h) < P-A (3.79 h) *** **- Screen time (television viewing, using a computer, playing other electronic games)**: P-C (2.21 h) < P-A (2.47 h) *** *Overall associations with sleep duration and disturbances* **- Daily outdoor time** showed a significantly positive association with bedtime and daytime sleep, and negative association with night-time sleep *** **- Screen time** showed a significantly positive association with bedtime, wake up time, sleep onset latency, number of night awakenings and night-time sleep *** ***Parental*** *Differences in parental factors between countries (Sleeping location)* **- Bedtime routine**: P-A (62.2%) < P-C (87.3%) **** **- Maternal education level**: No significant differences Elementary school (%): P-C (0.4) < P-A (0.5) High school (%): P-A (36.2) < P-C (37.5) College (%): P-C (43.0) < P-A (46.2) Postgraduate (%): P-A (17.1) < P-C (19.1) *Overall associations between bedtime routine and sleep duration and disturbances* **- The frequency of a consistent bedtime routine** showed significantly positive associations with night-time sleep and total sleep time whereas negative associations with bedtime, sleep onset latency, number of night awakenings, wake up time and daytime sleep were found *** ***Environmental*** *Differences in environmental factors between countries* **- Maternal employment status** **** Full time (%): P-C (27.7) < P-A (45.4) Part time (%): P-A (8.6) < P-C (21.6) Home/student (%): P-A (46.0) < P-C (50.7) **- Sleeping location** **Own room (%)**: P-A (10.1) < P-C (79.5) **** **Own bed (%)**: P-A (28.1) < P-C (90.6) **** *Controlling sleeping location examining differences in sleep duration and disturbances between countries* - Significant differences in sleep patterns between P-C and P-A countries were maintained after controlling for sleeping location, except for sleep onset latency.***
	P-C countries (*n* = 1139; AU, NZ, CA, US, UK)	3–6 years (mean not reported) /49.8%				
	P-A countries (*n* = 1447; CN, HK, IN, ID, KR, JP, MY, PH, SG, TW, TH, VN).	3–6 years (mean not reported) /50.2%				
Short et al. (2013) [35]	Australia (*n* = 385)	13–18 years (M = 15.57 y) / 60%	School start time, Extracurricular load, Circadian preference	Parent-set bedtime	not measured	***Child*** *Differences in child factors between countries* **- School Start time**: American (7:45 am) < Australian (8:32 am) *** **- Extracurricular load (hours)**: Australian (8.12) < US (13.4) *** Extracurricular load includes part-time work, sport, other activities and homework. **Part-time work (hours)**: Australian (1.51) < US (3.25) *** **Sport (hours)**: US (2.17) < Australian (2.27) **Other activities (hours)**: Australian (0.51) < US (1.54) *** **Homework (hours)**: Australian (4.17) < US (6.52) *** **- Circadian preference** (Smith Morningness/Eveningness Questionnaire score): Australian (33.25) < American (33.94) *Controlling countries examining the impact of child factors on sleep duration* **- School start time** had the largest effect on sleep duration *** For every hour earlier school start time, adolescents had an average of 29 min less sleep per night. This significant effect was disappeared after controlling for countries. **- Extracurricular load** was significantly associated with sleep duration per night.** Adolescents had 4 min less sleep per night with every hour spent per day on extracurricular activities. The significant relationship between extracurricular load and sleep duration was disappeared after controlling for countries.** ***Parental*** *Differences in parental factors between countries* **- Parent-set bedtime**: American (6.8%) < Australian (17.5%) *** *Controlling countries examining the impact of parental factors on sleep duration* - Adolescents gained 24 min of sleep per night if they had a parent-set bedtime. The significant relationship between parent-set bedtime and sleep duration was maintained after controlling for the country.**
	US (*n* = 302)	13–19 years (M = 16.03 y) /35%				
Chaput et al. (2015) [43]	12 countries (*n* = 5777) including	9–11 years	Diet, physical activity, screen time (i.e., hours of watching TV, playing video games and using computer)	not measured	not measured	***Child-related*** *Overall associations with sleep duration and disturbances* **- Healthy/unhealthy diet**: Unhealthy diet was associated with a later bedtime,*** a shorter sleep duration* and poorer sleep efficiency*** after controlling for covariates (e.g., age, gender, highest parental education and body mass index), but a healthy diet was only associated with an earlier bedtime.** The relationships were similar across sites, but not specified. **- Physical activity**: Moderate-to-vigorous physical activity was negatively associated with bedtime,**** sleep duration,**** and sleep efficiency **** after controlling for covariates **** Associations between sleep duration or disturbances (duration, efficiency and bedtime) and moderate-to-vigorous physical activity were different between study sites, but data are not shown. **- Screen time**: Screen time (i.e., hours of watching TV, playing video games and using a computer) was positively associated with bedtime after controlling for covariables (e.g., age, gender, highest parental education and body mass index) **** Screen time was also negatively associated with sleep quality, but the significant association was disappeared after controlling for covariables. No significant association was shown between screen time and nocturnal sleep duration. Associations between sleep duration or disturbances (duration, efficiency and bedtime) and screen time were different between study sites, but data were not shown.
	Australia (*n* = 433)	(M = 10.7 y)/46.7%				
	Canada (*n* = 496)	(M = 10.5 y)/40.9%				
	China (*n* = 459)	(M = 9.9 y)/51.6%				
	India (*n* = 433)	(M = 10.5 y)/45.1%				
	UK (*n* = 374)	(M = 10.9 y)/42.8%				
	US (*n* = 421)	(M = 9.9 y)/40.4%				
	Brazil (*n* = 435)	(M = 10.5 y)/48.5%				
	Colombia (*n* = 820)	(M = 10.5 y)/49.2%				
	Finland (*n* = 526)	(M = 10.4 y)/45.3%				
	Kenya (*n* = 452)	(M = 10.2 y)/45.4%				
	Portugal (*n* = 563)	(M = 10.4 y)/41.6%				
	South Africa (*n* = 452)	(M = 10.2 y)/38.6%				
Mindell et al. (2015) [44]	13 countries including AU/NZ, CA, UK, US, CN, HK, IN, JP, KR, MY, PH, SG and TH (*n* = 10085)	Birth–5.11 years (mean not reported)/49.6% (for entire sample)	not measured	Bedtime routine	not measured	***Parental*** *Differences in parental factors between countries* **- Bedtime routine**: P-A (64.4%) < P-C (82.6%) **** *Overall associations between bedtime routine and sleep disturbances* - Children with a consistent bedtime routine have earlier bedtimes, shorter sleep onset latency, more night-time sleep, fewer and shorter duration of night awakenings, and more total sleep per 24 h ***
Mindell et al. (2015) [45]	13 countries (*n* = 10085) including		not measured	Maternal education level, Maternal sleep	Number of children, Maternal employment	**Parental** *Differences in parental factors between countries* **- Maternal education level** **** Elementary school (%): P-A (0.4) < P-C (0.5) High school (%): P-A (33.5) < P-C (34.3) College (%): P-C (43.9) < P-A (49.1) Postgraduate (%): P-A (17.2) < P-C (21.4) *Overall association between maternal sleep and sleep duration and disturbances* **- Maternal sleep** was positively associated with children’s bedtime, wake up time, number of night awakenings and nocturnal sleep duration and the stronger relationship was shown in younger children *** ***Environmental*** *Differences in environmental factors between countries* **- The number of children**: No significant differences 1–P-A (53.9%) < P-C (54.3%) 2–P-A (32.6%) < P-C (33.7%) 3^+^–P-C (12.1%) < P-A (13.4%) **- Maternal employment status** **** Full time (%): P-C (27.0) < P-A (48.3) Part time (%): P-A (5.7) < P-C (17.6) Home/student (%): P-A (46.0) < P-C (55.4)
	P-C countries (*n* = 4152; AU/NZ, CA, UK, US) and	Birth–6 years (mean not reported)/50.7%				
	P-A countries (*n* = 5933; CN, HK, IN, JP, KR, MY, PH, SG and TH)	Birth–6 years (mean not reported)/50.2%				
Vazsonyi et al. (2015) [26]	Swiss (*n* = 5575)	15–18 years (M = 17.17)/ 50.0%	not measured	Parental warmth	not measured	***Parental*** *Associations between parental warmth and sleep duration and disturbances in each country* **- Both Swiss and Georgia**: Parental warmth had a significantly positive correlation with sleep quality and sleep quantity.
	Georgia (*n* = 6692)	15–18 years (M = 15.83)/ 40.0%				
Biggs et al. (2016) [37]	Australia (*n* = 87)	5–12 years (M = 9.6 y) /58%	not measured	Maternal education level	Season, Daylight amount	***Parental*** *Differences in parental factors between countries* **- Maternal education level < 11 Years schooling (%)**: No significant differences Australia (16) = Canada (16) High school Graduate (%): Australia (22) < Canada (27) College/University (%): Australia (22) < Canada (24) University Graduate (%): Canada (32) < Australia (37) Unknown (%): Australia (3) < Canada (1) ***Environmental*** *Differences in environmental factors between countries* **- Seasons of study**: No significant differences Spring: Canada (15%) < Australia (20%) Summer: Australia (23%) < Canada (26%) Fall: Australia (18%) < Canada (32%) Winter: Canada (28%) < Australia (39%) **- Daylight duration**: Australia (11:50) < Canada (11:55)
	Canada (*n* = 101)	5–12 years (M = 9.0 y) /56%				
Mindell et al. (2017) [38]	ME countries (*n* = 669; Saudi Arabia, Egypt, Algeria, United Arab Emirates, Jordan, Morocco, Iraq, Kuwai, Oman, Palestinian territories, Libyan Arab Jamahiriya, Bahrain, Israel and 83 from other Arab countries)	Birth–3 years (mean not reported) /50.2%	not measured	Bedtime routine	not measured	***Parental*** *Differences in parental factors between countries* **- Bedtime routine (5+ times/week)**: ME (46.79%) < PA (61.65%) < PC (71.13%) *** *Overall associations between bedtime routine and sleep duration and disturbances* - The frequency of a consistent bedtime routine had a significantly negative correlation with bedtime, sleep onset latency, number of night awakenings and duration of night awakenings, while positive correlation with nocturnal sleep and total sleep duration ***
	P-C and P-A countries from Mindell et al. (2010; *n* = 29,287) [15]	Birth–3 years (mean not reported) /51.94%				
Takahashi et al. (2018) [39]	Japan (*n* = 505)	4–5 years (mean not reported) /47.3%	not measured	Parental education level	Family structure, Number of siblings	***Parental*** *Differences in parental factors between countries* **- Maternal education level** *** Middle school or below (%): Japan (4.6) < China (13.9) High school (%): China (21.7) < Japan (74.4) Undergraduate or above (%): Japan (21.0) < China (64.4) **- Paternal education level** *** Middle school or below (%): Japan (5.9) < China (12.0) High school (%): China (20.8) < Japan (65.5) Undergraduate or above (%): Japan (28.6) < China (67.2) ***Environmental*** *Differences in environmental factors between countries* **- Family structure**: No significant differences Nuclear family: China (56.2%) < Japan (61.2%) Extended family: Japan (35.6%) < China (40.2%) Single parent or other: Japan (3.2%) < China (3.6%) **- The number of siblings** *** Only child: Japan (16.2%) < China (82.5%) Not the only child: China (17.5%) < Japan (83.8%)
	China (*n* = 1909)	4–5 years (mean not reported) /53.0%				
Carneiro et al. (2019) [28]	Cape Verde (*n* = 206)	2–15 years (mean not reported) /46%	Bedtime television	Parental education level	Number of cohabitants, number of cohabitant children	***Child*** *Associations between bedtime screen time and sleep disturbances in each country* **- Bedtime screen time (Fell asleep while watching TV)**: **Mozambique**: Bedtime screen time had a significantly positive association with children’s CSHQ scores*** showing that MZ children who “sometimes” watching TV at bedtime had approximately 12% higher CSHQ scores than children who “rarely” watching TV at bedtime. On the other hand, children who “usually” watch TV at bedtime had approximately 7% higher CSHQ scores than children who “rarely” watching TV at bedtime. **Cape Verde**: Significant association was not shown. ***Parental*** *Differences in parental factors between countries* **Mother education (<10 years, %)**: Mozambique (6%) < Cape Verde (74%) *** **Mother education (≥10 years, %)**: Cape Verde (26%) < Mozambique (94%) *** **Father education (<10 years, %)**: Mozambique (8%) < Cape Verde (68%) *** **Father education (≥10 years, %)**: Cape Verde (32%) < Mozambique (92%) *** *Associations between parental education level with sleep disturbances in each country* **- Maternal education level** **Cape Verde**: Mothers’ educational level had a significant association with the CSHQ scores, showing that children whose mothers have more than 9 years of education had approximately 7% lower CSHQ scores. **Mozambique**: Significant association was not shown. **- Paternal education level** **Mozambique**: Fathers’ educational level had a significant association with the CSHQ scores,* showing that children whose fathers have more than 11 years of education had approximately 4% lower CSHQ scores* than children whose fathers have less than 10 years of education. **Cape Verde**: Significant association was not shown. ***Environmental*** *Differences in family factors between countries* **- The number of cohabitant children** *** **(< 3)**: Cape Verde (58%) < Mozambique (80%) **(≥ 3)**: Mozambique (20%) < Cape Verde (42%) *** **- The number of cohabitants in family** *** **(< 5)**: Cape Verde (37%) < Mozambique (70%) **(≥ 5)**: Mozambique (30%) < Cape Verde (63%) *Associations between environmental factors with sleep disturbances in each country* **- The number of cohabitants children** **Both Cape Verde and Mozambique**: Number of cohabitant children did not have a significant association with CSHQ scores (overall sleep problems) **- The number of cohabitants in family** **Both Cape Verde and Mozambique**: Number of cohabitants in the family did not have a significant association with CSHQ scores (overall sleep problems)
	Mozambique (*n* = 438)	4–13 years (mean not reported) /49%				
Daban & Goh (2019) [40]	6 Southeast Asia countries (*n* = 5987) including	Birth–3 years	not measured	Bedtime routine	Sleeping location	***Parental*** *Differences in parental factors between countries* **Consistent bedtime routine**: PH (52.03) < ML (52.75) < SG (59.74) < ID (65.66) < VT (71) < TH (71.46) **** ***Environmental*** *Differences in environmental factors between countries* **- Sleeping location** **Own room** (%): TH (1.01) < VT (2.1) < PH (6.58) < ML (8.53) < ID (9.72) < SG (19.28) **** **Parent’s room** (%): SG (73.73) < ID (81.9) < ML (84.05) < PH (86.56) < VT (94.3) < TH (94.54) **** **Parent’s bed**: SG (35.86) < ML (44.03) < PH (65.09) < ID (70.73) < TH (77.23) < VT (83.2) ****
	Indonesia (*n* = 967)	(mean not reported) /50.2%				
	Malaysia (*n* = 997)	(mean not reported) /50.4%				
	Philippines (*n* = 1034)	(mean not reported) /49.8%				
	Singapore (*n* = 1001)	(mean not reported) /51.6%				
	Thailand (*n* = 988)	(mean not reported) /49.2%				
	Vietnam (*n* = 1000)	(mean not reported) /49.4%				
van Selms et al. (2019) [29]	Netherland (*n* = 1131)	7–12 years (M = 10.0) /44.6%	Pressure from home, Pressure from school, Easily scared, Worried	not measured	not measured	**Child** Differences in child factors between countries **- Pressure from home** (%): Indonesia (16.5) < The Netherlands (21.8) < Armenia (24.0) ** **- Pressure from school** (%): Indonesia (22.4) < The Netherland (23.9) < Armenia (31.4) ** **- Easily scared** (%): The Netherlands (27.0) < Indonesia (58.3) < Armenia (62.7) ** **- Worried** (%): The Netherland (46.7) < Armenia (49.3) < Indonesia (60.0) ** *Associations between child factors with sleep disturbances in each country* **- Pressure from home** **Netherland and Indonesia**: Children who experience pressure from the home situation, are more likely to have parental-reported SB. **Armenia**: Significant correlation was not shown **- Pressure from school** **All countries**: No association between pressure from school and parent-reported SB was shown. **- Easily scared** **Netherland and Armenia**: Children, who are easily scared, are more likely to have parent-reported SB. **Indonesia**: Significant correlation was not shown. **- Worried** **All countries**: No association between worried and parent-reported SB was shown.
	Armenia (*n* = 886)	7–12 years (M = 9.1) /49.0%				
	Indonesia (*n* = 545)	7–12 years (M = 9.5) /41.5%				
			7	17	13	

Note. M: Mean; y: years;. P-C: Predominantly-Caucasian; P-A: Predominantly-Asian; ME: Middle East; AU: Australia; CA: Canada; NZ: New Zealand; US: United States; UK: United Kingdom; CN: China; HK: Hong Kong; IN: India; ID: Indonesia; KR: Korea; JP: Japan; MY: Malaysia; PH: Philippines; SG: Singapore; TW: Taiwan; TH: Thailand; VN: Vietnam, CSHQ: Children’s Sleep Habits Questionnaire; SES: Socio-Economic Status; SB: Sleep Bruxism; * *p* < 0.05, ** *p* < 0.01, *** *p* < 0.001, **** *p* < 0.0001.
